# The LKB1–TSSK1B axis controls YAP phosphorylation to regulate the Hippo–YAP pathway

**DOI:** 10.1038/s41419-024-06465-4

**Published:** 2024-01-20

**Authors:** Cho-Long Kim, Su-Bin Lim, Sue-Hee Choi, Dong Hyun Kim, Ye Eun Sim, Eun-Hye Jo, Keeeun Kim, Keesook Lee, Hee-Sae Park, Su Bin Lim, Li-Jung Kang, Han-Sol Jeong, Youngsoo Lee, Carsten G. Hansen, Jung-Soon Mo

**Affiliations:** 1 Department of Biomedical Sciences, Graduate School, Ajou University School of Medicine, Suwon, 16499 South Korea; 2 School of Biological Sciences and Technology, Chonnam National University, Gwangju, 61186 South Korea; 3 Institute of Medical Science, Ajou University School of Medicine, Suwon, 16499 South Korea; 4 Department of Biochemistry and Molecular Biology, Ajou University School of Medicine, Suwon, 16499 South Korea; 5 Three-Dimensional Immune System Imaging Core Facility, Ajou University, Suwon, 16499 South Korea; 6 Division of Applied Medicine, School of Korean Medicine, Pusan National University, Yangsan, 50612 South Korea; 7 The University of Edinburgh, Institute for Regeneration and Repair, Centre for Inflammation Research, Edinburgh BioQuarter, Edinburgh, UK

**Keywords:** Cell growth, Phosphorylation, Oncogenes

## Abstract

The Hippo pathway’s main effector, Yes-associated protein (YAP), plays a crucial role in tumorigenesis as a transcriptional coactivator. YAP’s phosphorylation by core upstream components of the Hippo pathway, such as mammalian Ste20 kinase 1/2 (MST1/2), mitogen-activated protein kinase kinase kinase kinases (MAP4Ks), and their substrate, large tumor suppressor 1/2 (LATS1/2), influences YAP’s subcellular localization, stability, and transcriptional activity. However, recent research suggests the existence of alternative pathways that phosphorylate YAP, independent of these core upstream Hippo pathway components, raising questions about additional means to inactivate YAP. In this study, we present evidence demonstrating that TSSK1B, a calcium/calmodulin-dependent protein kinase (CAMK) superfamily member, is a negative regulator of YAP, suppressing cellular proliferation and oncogenic transformation. Mechanistically, TSSK1B inhibits YAP through two distinct pathways. Firstly, the LKB1–TSSK1B axis directly phosphorylates YAP at Ser94, inhibiting the YAP–TEAD complex’s formation and suppressing its target genes’ expression. Secondly, the TSSK1B–LATS1/2 axis inhibits YAP via phosphorylation at Ser127. Our findings reveal the involvement of TSSK1B-mediated molecular mechanisms in the Hippo–YAP pathway, emphasizing the importance of multilevel regulation in critical cellular decision-making processes.

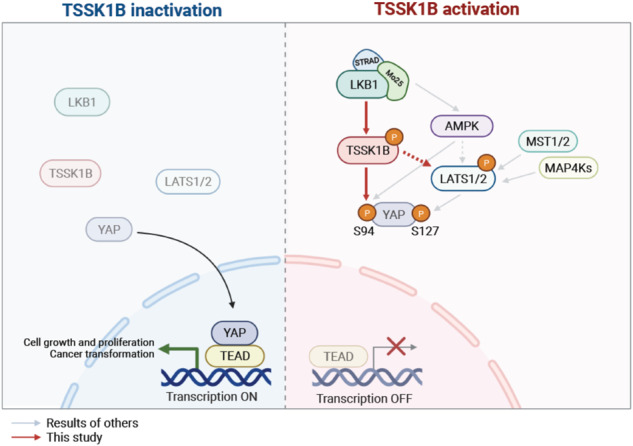

## Highlights

- TSSK1B directly phosphorylates Ser94 of YAP
- TSSK1B disrupts the interaction between YAP and TEAD, thereby inhibiting the transcriptional activity of YAP
- LKB1 is important for the activation of TSSK1B
- TSSK1B has an anti-cancer effect by impeding tumor growth

## Introduction

The Hippo pathway modulates tissue homeostasis and organ size, and its components are highly conserved from invertebrates to vertebrates [1]. Mutations of components or dysregulation of the Hippo pathway are associated with many human cancers [2]. The Ser/Thr kinases Mammalian Ste20 kinase 1/2 (MST1/2), mitogen-activated protein kinase kinase kinase kinases (MAP4Ks), and large tumor suppressor 1/2 (LATS1/2) are representative kinases that lie within a core kinase cascade [3]. They work with the adaptor proteins SAV and MOB to activate LATS1/2, which directly phosphorylates Yes-associated protein (YAP)/transcriptional coactivator with PDZ-binding motif (TAZ). The phosphorylation of the critical growth-promoting coactivators, YAP and TAZ, results in their sequestration within the cytoplasm and subsequent degradation via the proteasomal pathway [4, 5]. The phosphorylation status of YAP is a crucial determinant of its subcellular localization and binding partners, thereby significantly impacting its function as a transcriptional cofactor and other regulatory proteins [4, 6, 7].

Although the kinase cascade is the central component of the Hippo pathway, multiple upstream regulators can modulate the activity of YAP/TAZ [8]. The significance and the underlying mechanisms of several kinases to phosphorylate YAP, including NLK at Ser128 [9, 10], SRC family kinases (SFK) at Tyr357 [11, 12], and AMPK at Ser94 have been studied [13, 14]. Specifically, when AMPK is activated, it phosphorylates YAP at Ser94, preventing the formation of a hydrogen bond with the transcriptional enhanced associate domain (TEAD) and inhibiting the YAP–TEAD complex. This suppresses gene expression involved in cell proliferation and transformation [15–17]. Due to the favorable effects on growth mediated by YAP/TAZ, pathologically misregulated YAP/TAZ is commonly observed in various diseases, particularly cancer [18–20]. Studies using genetic mouse models have demonstrated that the loss of Hippo core components, which regulate YAP/TAZ activity, leads to overgrowth phenotypes and ultimately contributes to cancer development [21, 22]. Therefore, it is essential to understand better the regulators influencing YAP/TAZ activity to unravel the intricate mechanisms underlying tumorigenesis.

In this study, we screened a sub-kinome library to identify genes that phosphorylate YAP and found that TSSK1B, a member of the testis-specific serine/threonine-protein kinase (TSSK) family, was identified as a potent negative regulator of YAP activity. The members of the TSSK family, including Tssk1 and Tssk2, show unique expression patterns during spermatogenesis in the testis [23–26]. However, we found that the mRNA and protein of TSSK1B are also expressed in other types of cells, suggesting that TSSK1B expression may not be restricted to testis.

Here we show that TSSK1B acts as an upstream activator of LATS1/2 kinase to phosphorylate Ser127 of YAP. Additionally, TSSK1B directly phosphorylates YAP Ser94, a critical TEAD binding residue [16]. Moreover, downregulation of *TSSK1B* expression through shRNAs or CRISPR/Cas9-mediated knockout results in nuclear YAP accumulation and the induction of YAP–TEAD target genes. TSSK1B efficiently antagonizes YAP activity even in cells with abnormally high proliferative capacity. Further detailed mechanistic studies demonstrate that LKB1 is a TSSK1B activating kinase, which stimulates TSSK1B kinase activity toward YAP. These data provide evidence that TSSK1B negatively regulates tumorigenesis through YAP inhibition.

## Results

### TSSK1B induces YAP phosphorylation and inhibits its transcriptional activity

**Fig. 1:**
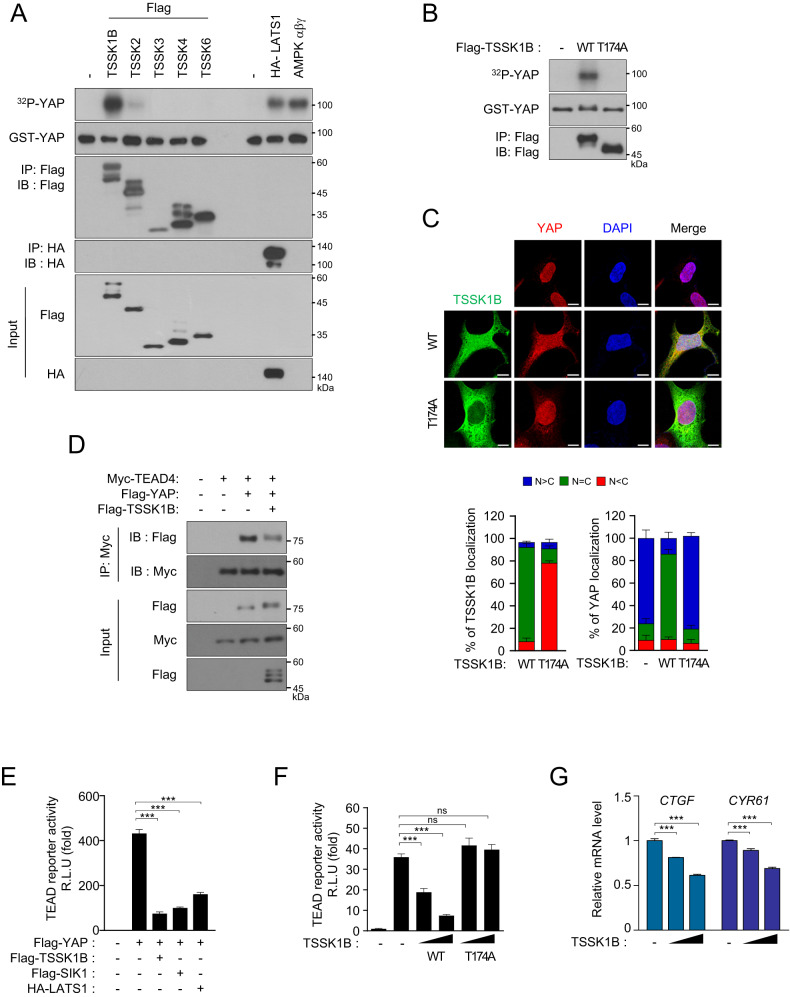
TSSK1B inhibits the transcriptional activity of YAP through phosphorylation. **A** Flag-TSSK kinase members and HA-LATS1 were expressed in HEK293A cells, and immunoprecipitated kinases and purified AMPKαβγ were used for the kinase reaction with the substrate GST-YAP prepared from *Escherichia coli*. Phospho-YAP (pYAP) was analyzed using ^32^P autoradiography, and GST-YAP was confirmed by immunoblotting. - : empty vector for control. **B** Flag-TSSK1Β wild-type (WT) or mutant (T174A) construct was immunoprecipitated using anti-Flag Ab. Immunoprecipitated kinases were used for the kinase reaction with GST-YAP. pYAP was analyzed using ^32^P autoradiography. **C** HEK293A cells were transiently transfected with Flag-TSSK1B WT or mutant (T174A) construct and were subjected to immunofluorescence staining for TSSK1Β (green) and YAP (red); 4’,6-diamidino-2-phenylindole (DAPI) (blue) was used for nuclear staining. The nuclear (N)–cytoplasmic (C) ratio of YAP was analyzed in three randomly selected fields from three independent experiments (n = 100 cells per field). Scale bars, 10 µm. **D** Flag-TSSK1B along with Flag-YAP and Myc-TEAD4 were expressed in HEK293A cells, and Myc-TEAD4 was immunoprecipitated using anti-Myc Ab followed by immunoblotting. **E** Constructs of different kinases were co-transfected with YAP, GAL4-TEAD4, 5xUAS luciferase reporter, or Renilla into HEK293A cells. After 48 h, the luminescent signal controlled by YAP–TEAD was measured and normalized to Renilla luciferase activity. Error bars indicate the standard error of the mean (SEM) from three separate points in one experiment (*n* = 3). ***p < 0.001. Student’s *t*-test was used for statistical analysis. **F** Flag-TSSK1B WT or mutant (T174A) construct was co-transfected with YAP, GAL4-TEAD4, 5xUAS luciferase reporter, or Renilla into HEK293A cells. After 48 h, the luminescent signal controlled by YAP–TEAD was measured and normalized to Renilla luciferase activity. Error bars indicate mean ± SEM (*n* = 3). ***p < 0.001. NS: Not significant. Student’s *t*-test was used for statistical analysis. **G** HEK293A cells were transiently transfected with the indicated plasmid. The mRNA levels of *CTGF* and *CYR61* were determined using quantitative real-time polymerase chain reaction (qRT-PCR) and normalized to *hypoxanthine-guanine phosphoribosyltransferase 1* (*HPRT1*) mRNA levels. Error bars indicate mean ± SEM (*n* = 3). ***p < 0.001. Student’s *t*-test was used for statistical analysis.

Previous reports highlighted the important role of AMPK in phosphorylating YAP at multiple serine residues, thereby regulating its function as a transcriptional cofactor [13, 14]. These findings hold significance as it provides a crucial strategy to develop anticancer drugs for cancers with dysregulated Hippo signaling. To identify potential kinase(s) of YAP, we screened the CAMK superfamily members that share structural similarities with AMPK. In our in vitro kinase assay using YAP as a substrate and 19 members of the CAMK superfamily as a potential kinase(s), TSSK1B was identified to phosphorylate YAP effectively (Fig. 1A and S1A). The kinase activity of TSSK1B on YAP was found to be more potent than that of LATS1 or AMPKαβγ (Fig. 1A).

As a member of the CAMK kinase family, TSSK1B possesses a T-loop threonine that is highly conserved and whose threonine phosphorylation is essential for full kinase activity (Fig. S1B). To determine the importance of T-loop threonine (Thr174) of TSSK1Β in YAP regulation, we generated a mutant by replacing Thr174 with alanine in the catalytic domain. We found that TSSK1Β wild-type (WT), but not its mutant (T174A), shows high kinase activity toward YAP phosphorylation (Fig. 1B). Next, we examined whether TSSK1B regulates the subcellular localization of YAP. While the expression of TSSK1B WT induced a cytoplasmic localization of YAP, the TSSK1B mutant (T174A) could not induce YAP cytoplasmic localization (Fig. 1C). These results suggest that TSSK1B-mediated phosphorylation of YAP effectively alters the subcellular localization of YAP. As expected, TSSK1B inhibits the YAP–TEAD interaction (Fig. 1D). To further establish the effect of TSSK1B on the YAP–TEAD complex, the transcriptional activity of YAP–TEAD was determined using a luciferase reporter assay. We found that TSSK1B robustly suppressed the transcriptional activity of TEAD as efficiently as did LATS1 and SIK1, known as YAP kinases, which are known to phosphorylate YAP to inhibit its activity (Fig. 1E) [4, 27]. In addition, the transcriptional activity of the TEAD luciferase reporter was significantly decreased in cells expressing TSSK1Β WT but not TSSK1Β mutant (T174A) (Fig. 1F). Consistent with the role of TSSK1B in YAP inhibition, TSSK1B notably decreased the mRNA levels of the YAP–TEAD targets, *CTGF* and *CYR61* [15, 28], thereby reflecting its inhibition of YAP–TEAD activation (Fig. 1G). Collectively, these results highlight that TSSK1Β kinase activity inhibits YAP.

### Endogenous TSSK1B attenuates the nuclear localization of YAP and suppresses its transcriptional activity

**Fig. 2:**
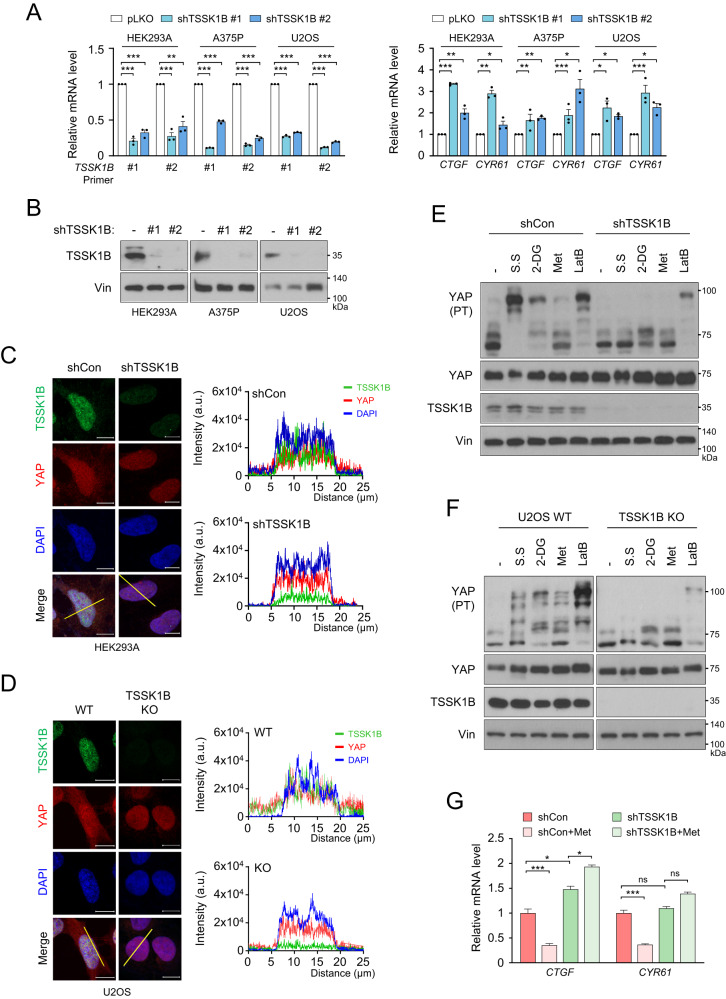
Deletion of TSSK1B reduces YAP phosphorylation and elevates YAP activity. **A** HEK293A, A375P, and U2OS cells were treated with shCon and shTSSK1B and qRT-PCR was performed using specific primer pairs to quantify the mRNA levels of *TSSK1B*, *CTGF*, and *CYR61*. The measurements were normalized with respect to the *HPRT1* mRNA levels. Error bars depict mean ± SEM (*n* = 3). ***p < 0.001. **p < 0.01. *p < 0.05. Student’s *t*-test was used for statistical analysis. **B** HEK293A, A375P, and U2OS cells were transduced using shCon or shTSSK1B, and knockdown efficiency for *TSSK1B* was confirmed by immunoblotting. Vin: Vinculin. **C** HEK293A cells treated with shCon or shTSSK1B were subjected to immunofluorescence staining for TSSK1Β (green) and YAP (red); 4’,6-diamidino-2-phenylindole (DAPI) (blue) was used for nuclear staining. Scale bars, 10 µm. The fluorescence intensity of TSSK1B, YAP, and DAPI was quantified by drawing a line (yellow) of 25 μm in a merged figure using the ZEN 3.5 blue edition program. **D** Knockout of *TSSK1B* using CRISPR/Cas9 in U2OS cells was confirmed by immunocytochemistry. Scale bars, 10 µm. The fluorescence intensity of TSSK1B, YAP, and DAPI was quantified by drawing a line (yellow) of 25 μm in a merged figure using ZEN 3.5 blue edition program. **E** HEK293A cells treated with shCon and shTSSK1B were incubated with serum-free medium (S.S) for 2 h, 25 mM 2-Deoxy glucose (2-DG) for 2 h, 1 mM metformin (Met) for 4 h, or 0.25 μg·ml^−1^ latrunculin B (LatB) for 30 min, and whole cell lysates were immunoblotted. PT: phos-tag gel. **F** U2OS WT and *TSSK1B* KO cells were incubated with serum-free medium (S.S) for 2 h, 25 mM 2-Deoxy glucose (2-DG) for 2 h, 1 mM metformin (Met) for 4 h, or 0.25 μg·ml^−1^ latrunculin B (LatB) for 30 min. Whole-cell lysates were immunoblotted. PT: phos-tag gel. **G** shCon- or shTSSK1B-treated HEK293A cells were incubated with 1 mM metformin for 4 h. The mRNA levels of *CTGF* and *CYR61* were determined. The relative unit values for mRNA were normalized to one with respect to that of the control group without metformin treatment. Error bars depict mean ± SEM (*n* = 3). ***p < 0.001. *p < 0.05. NS: Not significant. Student’s *t*-test was used for statistical analysis.

Although TSSK1B expression is known to be restricted to testis [29], we attempted to analyze its expression in different model systems. Our qRT-PCR using *TSSK1B* gene-specific primer sets and western blotting analysis using the antibody against TSSK1B revealed that TSSK1B is also expressed in the readily available cell lines, including in HEK293A, A375P, and U2OS cells at mRNA and protein levels (Figs. 2A, B, and S2A). In addition, the knockdown of the *TSSK1B* gene using two short hairpin RNAs (shRNAs) efficiently silenced the expression of TSSK1B at the mRNA and protein levels in HEK293A, A375P, and U2OS cells (Figs. 2A, B, and S2A). *TSSK1B* knockdown in these cell lines led to upregulated mRNA levels of two YAP–TEAD target genes, *CTGF* and *CYR61*, as shown in Fig. 2A [15, 28]. Immunofluorescence staining demonstrated that endogenous TSSK1B was more enriched in the nucleus rather than in the cytoplasm, while YAP was distributed in both the nucleus and cytoplasm (Figs. 2C and S2B). Knockdown of *TSSK1B* considerably decreased the expression of YAP in the cytoplasm but not in the nucleus, indicating that TSSK1B depletion alters the cellular localization of YAP. In addition to shRNA-mediated knockdown, we generated *TSSK1B* knockout (KO) cell lines in U2OS cells using CRISPR/Cas9-mediated gene editing [30]. We obtained heterozygous deletion clone (#1) and complete deletion clone (#2) of the *TSSK1B* gene and confirmed the successful generation (creation) of *TSSK1B* KO cell lines through western blotting (Figs. S3A, B). While YAP was distributed in both the nucleus and cytoplasm of TSSK1B WT U2OS cells, the deletion of *TSSK1B* exhibited an increased nuclear expression of YAP (Fig. 2D). These results suggest that the role of TSSK1B may not be restricted to spermatogenesis and sperm differentiation [24] and may act as a negative regulator of the Hippo pathway through the phosphorylation of YAP.

We next evaluated whether TSSK1B is involved in YAP phosphorylation in response to the signals that stimulate the Hippo pathway. As expected, treatment with the stresses, including serum starvation, 2-Deoxy glucose (2-DG), metformin (Met), or latrunculin B (LatB), induced YAP phosphorylation in HEK293A cells, showing multiple protein band patterns in phos-tag gel (Figs. 2E and S4A). Notably, *TSSK1B* knockdown in A375P cells and *TSSK1B* KO in U2OS cells potently reduced YAP phosphorylation induced by these stresses (Figs. 2F and S4B). These results suggest that endogenous TSSK1B plays a crucial role in this stress-induced YAP phosphorylation. Additionally, the mRNA levels of *CTGF* and *CYR61* were decreased in metformin-treated cells, but not in *TSSK1B*-deficient cells (Figs. 2G and S4C). *TSSK1B* KO in U2OS cells consistently, increased the basal *CTGF* mRNA levels and blocked the metformin-mediated downregulation of *CTGF* mRNA levels (Fig. S4D). Therefore, TSSK1B is likely involved in relaying the stress signal to regulate YAP activity through phosphorylation.

### LKB1 is required for TSSK1B-mediated YAP phosphorylation

**Fig. 3:**
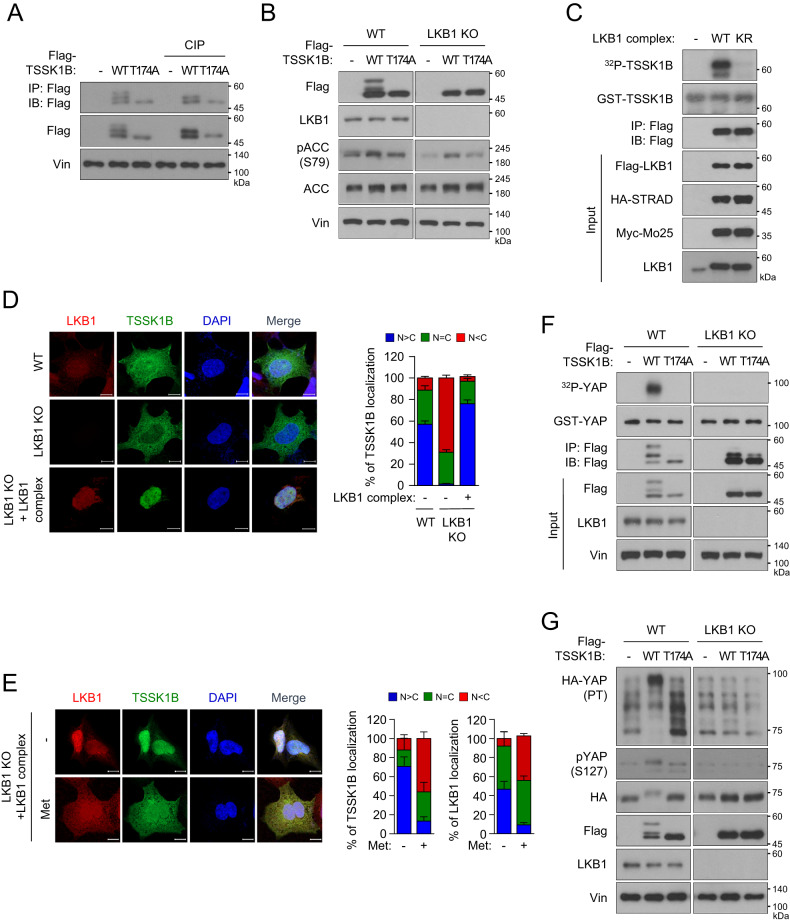
Identification of LKB1 as the upstream activator of TSSK1B. **A** Immunoprecipitated TSSK1B was treated with calf intestinal alkaline phosphatase (CIP) and detected by immunoblotting. The upper bands disappear after CIP treatment indicating that they represent phosphorylated proteins. Vin: Vinculin. **B** Cell lysates from HEK293A WT or *LKB1* KO cells expressing Flag-TSSK1B WT, or mutant (T174A) were subjected to immunoblotting. ACC: Acetyl CoA carboxylase. **C** Flag-LKB1 WT or KR, HA-STRAD, and Myc-Mo25 were expressed in HEK293A cells and immunoprecipitated LKB1 kinases were used for the kinase reaction with GST-TSSK1B. pTSSK1B was analyzed using ^32^P autoradiography, and GST-TSSK1B was detected by immunoblotting. **D** Flag-TSSK1B or Flag-LKB1 along with HA-STRAD and Myc-Mo25 were expressed in HEK293A WT and *LKB1* KO cells, whereafter they were subjected to immunofluorescence assay. The nuclear (N)–cytoplasmic (C) ratio of TSSK1B was analyzed in three randomly selected fields from three independent experiments (n = 100 cells per field). Scale bars, 10 µm. **E** HEK293A *LKB1* KO cells were transiently transfected using Flag-TSSK1B or LKB1 along with HA-STRAD and Myc-Mo25 and treated with 1 mM Metformin for 4 h. Cells were subjected to immunofluorescence assay. The nuclear (N)–cytoplasmic (C) ratios of TSSK1B and LKB1 were analyzed in three randomly selected fields from three independent experiments (n = 30 cells per field). Scale bars, 10 µm. **F** Flag-TSSK1B WT or mutant (T174A) was expressed in HEK293A WT and *LKB1* KO, and immunoprecipitated TSSK1B kinases were used for the kinase reaction with GST-YAP. pYAP was analyzed using ^32^P autoradiography, and GST-YAP was detected by immunoblotting. **G** HEK293A WT and *LKB1* KO cells were transfected with Flag-TSSK1B WT or mutant (T174A) along with HA-YAP, and whole cell lysates were immunoblotted. PT: phos-tag gel.

Next, we investigated the detailed regulatory mechanism by which TSSK1B inhibits YAP. Interestingly, ectopically expressed Flag-TSSK1B showed three protein bands with an increase of up to 15 kDa, suggesting the possibility of post-translational modification of TSSK1B (Fig. 1A). As protein kinases are known to be regulated through phosphorylation, either by themselves or by other kinases, we attempted to test whether the band shift of TSSK1B was due to phosphorylation. Treatment with calf intestinal alkaline phosphatase (CIP) eliminated the slow-migrating protein bands, confirming that the band shift of TSSK1B was indeed due to phosphorylation. The slow-migrating bands also disappeared in the TSSK1B mutant (T174A) (Fig. 3A), suggesting that phosphorylation of TSSK1B may be associated with its activation as a kinase. These results are consistent with the previous report that TSSKs autophosphorylate their Thr residue [31, 32]. Next, we tested whether upstream kinases could regulate TSSK1B phosphorylation in addition to autophosphorylation.

LKB1, a known master kinase belonging to the complex composed of STRAD and Mo25, phosphorylates Thr residue in the T-loop and activates 13 kinases of the AMPK subfamily [33, 34]. It was previously suggested that LKB1 might not be a major kinase for the TSSK family [31, 35]. Although TSSKs undergo autophosphorylation at the Thr residue [31, 32], we could not completely rule out the possibility that upstream kinases, such as LKB1, may also regulate TSSK1B phosphorylation on Thr in the T-loop (Fig. S1B) [36]. To examine this possibility, we compared the phosphorylation status of exogenously overexpressed TSSK1B WT with that of the corresponding mutant (T174A) in HEK293A WT and *LKB1* KO cells. We noticed a considerable band shift of exogenous TSSK1Β in WT, but not in *LKB1* KO HEK293A cells; however, we did not detect any band shift of TSSK1B mutant (T174A) in both HEK293A WT and *LKB1* KO cells (Figs. 3B and S5A). This result shows that LKB1 is required for the mobility shift of TSSK1Β protein band. Reconstitution of LKB1 complex (LKB1–STRAD–Mo25) in *LKB1* KO cells restored TSSK1Β mobility shift, suggesting that functional LKB1 complex can induce TSSK1Β phosphorylation (Fig. S5B). Exogenous LKB1 WT complex strongly phosphorylated TSSK1Β WT, whereas LKB1 kinase-inactive (KR) complex did not (Fig. 3C). Subsequently, through cell fractionation to separate cytoplasm and nucleus, we detected the phosphorylation of TSSK1B by LKB1 specifically in the nucleus (Fig. S5C). Utilizing immunocytochemistry, we observed that the distribution of overexpressed TSSK1B was ubiquitous throughout the cells (Fig. 3D). However, *LKB1* deletion induced a significant cytoplasmic localization of TSSK1Β, which was reversed upon expression of the LKB1 complex. These results suggest that LKB1 co-localizes with TSSK1B, affecting its cellular localization. Next, we evaluated whether metabolic stress-activated LKB1 affects the subcellular localization of TSSK1B in cells that lack LKB1. Immunofluorescence microscopy revealed that overexpressed LKB1 was mainly localized in the nucleus in untreated cells, but metformin treatment remarkably increased the cytoplasmic localization of LKB1 (Fig. 3E) [33, 37]. While LKB1 and TSSK1Β co-localized in the nucleus of resting cells at steady state, metformin treatment induced LKB1-mediated translocation of TSSK1Β from the nucleus to the cytoplasm, confirming a critical role of the LKB1 complex in controlling the subcellular localization of TSSK1Β. Therefore, LKB1 plays an essential role in promoting TSSK1B phosphorylation and activation.

As LKB1 phosphorylates TSSK1B and affects its localization, we next investigated whether LKB1 affects TSSK1B-mediated YAP phosphorylation. TSSK1Β immunoprecipitated from HEK293A WT cells displayed a high kinase activity toward YAP, but TSSK1B immunoprecipitated from *LKB1* KO cells did not (Figs. 3F and S5D). Expression of TSSK1B WT, but not that of the mutant (T174A), significantly induced YAP phosphorylation, as indicated by the altered mobility shift of YAP in phos-tag gel (Fig. 3G). Notably, TSSK1Β failed to induce a robust shift in YAP mobility in *LKB1* KO cells, highlighting the requirement of LKB1 for TSSK1B activity to phosphorylate YAP. These results suggest that the LKB1 complex may play an essential role in the subcellular localization of TSSK1B and its phosphorylation-mediated kinase activity. In conclusion, our investigation into the mechanism by which TSSK1B exhibits high kinase activity toward YAP, and this activity was dependent on the presence of LKB1.

### TSSK1B induces YAP phosphorylation through LATS1/2 in a MST1/2- and MAP4Ks-dependent manner

**Fig. 4:**
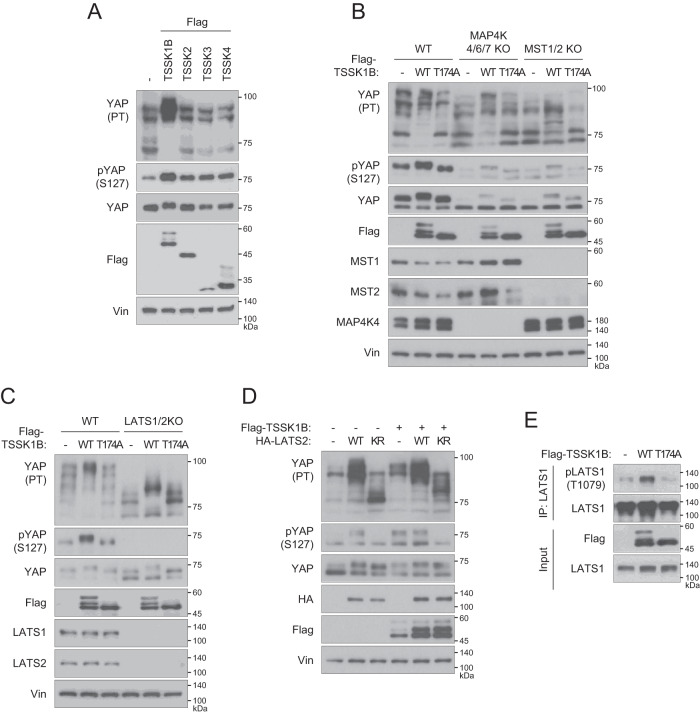
TSSK1B regulates YAP phosphorylation through the Hippo pathway. **A** Cell lysates from HEK293A cells expressing Flag-TSSKs were subjected to immunoblotting. YAP phosphorylation status was analyzed using a phos-tag gel to assess the phosphorylation-dependent mobility shift. Vin: Vinculin. PT: phos-tag gel. **B** HEK293A WT, *MAP4K4/6/7* KO, and *MST1/2* KO cells were transiently transfected with Flag-TSSK1B WT or mutant (T174A) construct and whole cell lysates were immunoblotted. PT: phos-tag gel. **C** HEK293A WT and *LATS1/2* KO cells were transfected with the indicated plasmids and whole cell lysates were immunoblotted. PT: phos-tag gel. **D** HEK293A cells were transfected with empty vector (-), Flag-TSSK1B, LATS2 WT, or LATS2 KR mutants. Whole cell lysates were immunoblotted using the indicated Abs. PT: phos-tag gel. **E** Flag-TSSK1B WT or mutant (T174A) construct was expressed in HEK293A cells and immunoprecipitated using anti-LATS1 Ab. LATS1 phosphorylation levels were detected by immunoblotting using anti-pLATS1 Thr1079 Ab.

The altered robust mobility shift of YAP on phos-tag gel indicates TSSK1B-induced YAP phosphorylation at multiple sites, including Ser127 (Fig. 4A). This observation encouraged us to examine the crosstalk between TSSK1B and the Hippo–YAP signaling. It is well-characterized that MAP4Ks/MST1/2–LATS1/2 axis activated by various cellular stimuli is involved in the phosphorylation of YAP at Ser127, yielding decreased cell proliferation and survival [38, 39]. To explore whether TSSK1B-mediated Ser127 phosphorylation of YAP relies on the MAP4Ks/MST1/2–LATS1/2 pathway, we expressed TSSK1Β WT and mutant (T174A) in HEK293A WT, *MST1/2* KO, *MAP4K4/6/7* KO, and *LATS1/2* KO cells. Our results show that TSSK1B induced a significant mobility shift of phosphorylated YAP protein bands, leading to higher molecular weight in phos-tag gel (Fig. 4B). These results suggest that YAP may be phosphorylated at multiple sites in the presence of TSSK1B. Additionally, TSSK1B WT expression significantly enhanced YAP phosphorylation at Ser127 in WT cells, whereas this effect was partially reduced in *MAP4K4/6/7* KO or *MST1/2* KO cells (Fig. 4B). These results suggest that MST1/2 or MAP4Ks partially regulate TSSK1B-mediated YAP phosphorylation. Notably, TSSK1Β-induced Ser127 phosphorylation of YAP was completely abolished in *LATS1/2* KO cells, indicating that TSSK1B induces Ser127 phosphorylation of YAP through LATS1/2 (Fig. 4C). Additionally, the LATS2 kinase-inactive (KR) mutant attenuated the TSSK1B-induced mobility shift of YAP, suggesting a dependence on LATS1/2 kinase activity (Fig. 4D). Furthermore, we observed that TSSK1B, but not the mutant (T174A), prominently induced phosphorylation of the hydrophobic motif Thr1079 of LATS1, leading to LATS1 activation (Fig. 4E). The phosphorylation of LATS1 at Ser909 and Thr1079 is known to positively correlate with its kinase activity [38, 40, 41]. These findings suggest that TSSK1Β integrates the Hippo pathway by enhancing LATS1/2 kinase activity in a MAP4Ks- and MST1/2-dependent manner.

### TSSK1B directly phosphorylates YAP at Ser94 to regulate its activity

**Fig. 5:**
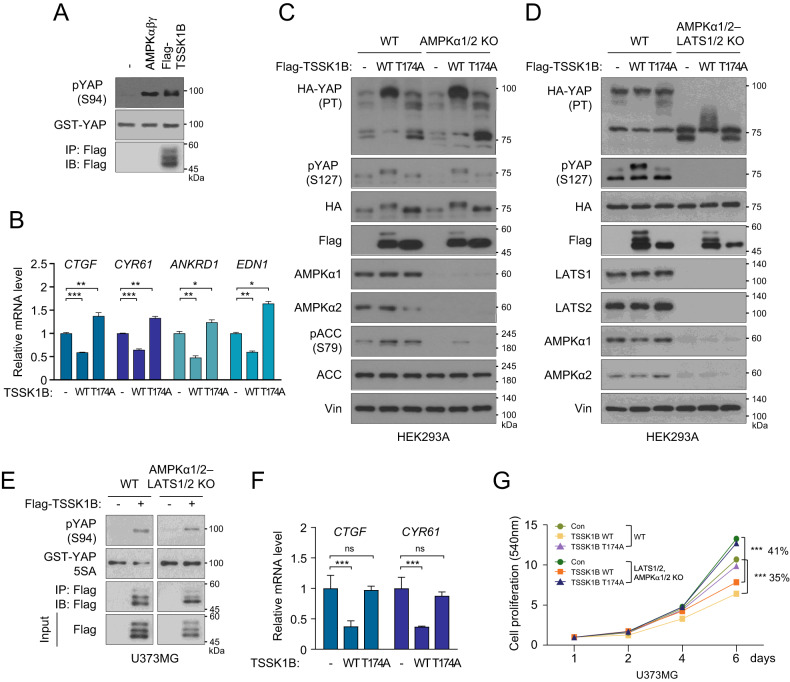
TSSK1B phosphorylates YAP at Ser94, negatively affecting its transcriptional activity and cell proliferation. **A** Immunoprecipitated Flag-TSSK1B and purified AMPKαβγ complexes were used for the kinase reaction with GST-YAP. YAP phosphorylation was analyzed by immunoblotting. **B** HEK293A *LATS1/2* KO cells were transiently transfected with the indicated plasmids. qRT-PCR determined the mRNA levels of *CTGF, CYR61, ANKRD1*, and *END1*. Error bars represent mean ± SEM (*n* = 3). ***p < 0.001. **p < 0.01. *p < 0.05. Student’s *t*-test was used for statistical analysis. **C** HEK293A WT and *AMPKα1/2* KO cells were transiently transfected with an empty vector (-), Flag-TSSK1B WT, or mutant (T174A) construct, and whole cell lysates were immunoblotted. YAP phosphorylation status was analyzed using a phos-tag gel. PT: phos-tag gel. Vin: Vinculin. **D** HEK293A WT and *AMPKα1/2–LATS1/2* KO cell pools were transiently transfected with the indicated plasmids, and whole cell lysates were immunoblotted. YAP phosphorylation status was analyzed using a phos-tag gel. PT: phos-tag gel. **E** U373MG WT and *AMPKα1/2–LATS1/2* KO cell pools were transfected with Flag-TSSK1B WT and immunoprecipitated using anti-Flag Ab. Immunoprecipitated Flag-TSSK1B was used with GST-YAP 5SA prepared from *E. coli*. YAP phosphorylation and GST-YAP 5SA were detected by immunoblotting using anti-pYAP Ser94 or anti-GST Abs, respectively. **F** U373MG WT and *AMPKα1/2–LATS1/2* KO cell pools were transiently transfected with the indicated plasmids. qRT-PCR determined the mRNA levels of *CTGF* and *CYR61*. Error bars represent mean ± SEM (*n* = 3). ***p < 0.001. NS: Not significant. Student’s *t*-test was used for statistical analysis. **G** pBABE-vector (Con), pBABE-TSSK1Β WT, or mutants (T174A) were stably expressed in U373MG WT and *AMPKα1/2–LATS1/2* KO cell pools, and cell proliferation was measured using SRB assay. Values represent mean ± SEM (*n* = 3). ***p < 0.001. Student’s *t*-test was used for statistical analysis.

Examining YAP’s total phosphorylation patterns using phos-tag gel revealed a significant mobility shift into multiple protein bands with lower molecular weight, suggesting potential LATS1/2-independent YAP phosphorylation by TSSK1B (Fig. 4C). This result indicates that TSSK1Β may contribute to YAP phosphorylation at sites other than Ser127, independent of LATS1/2 signaling. Previously, we showed that Ser94 phosphorylation of YAP by AMPK hampers hydrogen bond formation with TEAD, thereby inhibiting YAP–TEAD transcriptional activity [13]. To confirm that TSSK1B directly phosphorylates YAP at Ser94, we used a phospho-specific antibody for pYAP Ser94. TSSK1B phosphorylates full-length and truncated YAP (51–270 a.a), but not the YAP S94A mutant, suggesting that the Ser94 residue is the main direct YAP target site of TSSK1B (Figs. 5A and S6A). Hence, we tested whether TSSK1B negatively regulates YAP, and we measured the mRNA levels of the YAP–TEAD target genes, even in the absence of *LATS1/2*. qRT-PCR analysis reveals that TSSK1B WT, but not the mutant (T174A), decreased *CTGF, CYR61, ANKRD1*, and *END1* mRNA levels in HEK293A *LATS1/2* KO cells (Fig. 5B). Additionally, the protein expression of CTGF and CYR61 exhibited a similar pattern (Fig. S6B).

Activation of AMPK leads to YAP inhibition by two distinct mechanisms, (i) direct inhibition via phosphorylating YAP and (ii) indirectly via activating LATS1/2 kinase [13, 14, 42, 43]. To investigate the role of AMPKα1/2 in TSSK1Β-induced YAP phosphorylation, we generated HEK293A *AMPKα1/2* KO and HEK293A *AMPKα1/2–LATS1/2* KO cells (Fig. S6C). Notably, YAP phosphorylation at Ser127 by the ectopic expression of TSSK1Β WT, but not that of TSSK1Β mutant (T174A), was similarly observed in both HEK293A WT and *AMPKα1/2* KO cells (Fig. 5C). A further experiment using *AMPKα1/2–LATS1/2* KO HEK293A cells also revealed that TSSK1Β-induced YAP phosphorylation at Ser127 is completely abolished in these cells (Fig. 5D). However, TSSK1Β WT-dependent phosphorylation with lower molecular weight than that in HEK293A WT cells was observed in the absence of *AMPKα1/2* and *LATS1/2*. These results indicate that TSSK1Β-mediated phosphorylation of YAP at Ser127 depends on LATS1/2. Additionally, TSSK1B can phosphorylate YAP at Ser94 independently of AMPK, suggesting its ability to regulate YAP activity regardless of AMPKα1/2 and LATS1/2.

To investigate the impact of TSSK1B on cell proliferation and colony formation, we created *AMPKα1/2–LATS1/2* KO U373MG cell lines (Fig. S6D). We first ectopically introduced TSSK1B into these cells and then immunoprecipitated TSSK1B from U373MG WT and *AMPKα1/2–LATS1/2* KO cells. The results show that the kinase activity of TSSK1B towards YAP is comparable in both cell lines (Fig. 5E). Moreover, exogenous TSSK1B expression decreased the mRNA levels of *CTGF* and *CYR61* in U373MG *AMPKα1/2–LATS1/2* KO cells (Fig. 5F). We further observe that TSSK1B suppresses cell proliferation, whereas TSSK1B mutant (T174A)-expressing cells exhibit higher proliferation than cells transfected with the TSSK1B WT. Additionally, *AMPKα1/2–LATS1/2* KO cells markedly increased the proliferation of U373MG cells compared to that of WT cells. Cells expressing TSSK1B WT exhibited an anti-proliferative effect, whereas cells expressing TSSK1B mutant (T174A) proliferated similarly to the empty vector control (Fig. 5G). Our results suggest that TSSK1B can regulate YAP/TAZ independently of LATS1/2 or AMPKα1/2.

### TSSK1B acts to inhibit the tumorigenic potential of YAP independent of LATS1/2

**Fig. 6:**
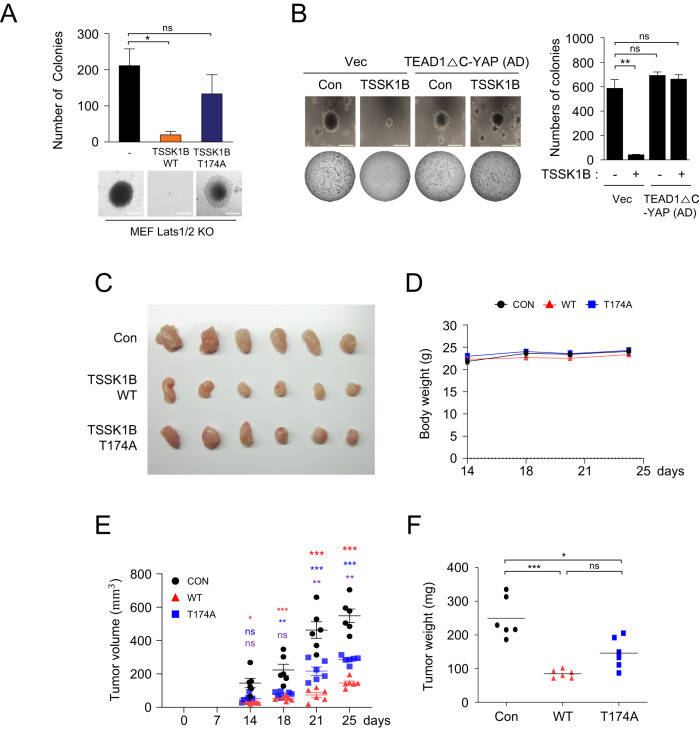
TSSK1B suppresses anchorage-independent growth and inhibits tumor growth independent of LATS1/2. **A** MEF *Lats1/2* KO cells expressing pBABE-vector (-), TSSK1B WT, or mutant (T174A) were assessed for colony formation in soft agar. The representative colony formations were stained with crystal violet. Values represent mean ± SEM (*n* = 3). *p < 0.05. NS: Not significant. Student’s *t*-test was used for statistical analysis. Scale bars, 200 µm. **B** MEF *Lats1/2* KO cells expressing pBABE-vector, pBABE-TSSK1B, pPGS-HA-vector, and pPGS-HA-TEAD1ΔC–YAP(AD) were assessed for colony formation in soft agar. MEF *Lats1/2* KO expressing TSSK1B exhibited an increase in soft agar colony formation. Error bars depict mean ± SEM (*n* = 3). **p < 0.01. NS: Not significant. Student’s *t*-test was used for statistical analysis. Scale bars, 200 µm. **C**–**F** TSSK1B inhibits tumor growth. **C** The image of the tumors dissected from BALB/c nude mice with pBABE-vector (Con), TSSK1Β WT, or mutant (T174A) in MEF *Lats1/2* KO cells on the 25th day after injection. **D** In the three groups, the mice’s body weights did not differ significantly. **E** The tumor growth in the TSSK1B WT xenograft group was significantly lower than that in the control and mutant (T174A) groups. *P*-value: Con vs. WT (red), Con vs. mutant (T174A) (blue), WT vs. mutant (T174A) (purple). Error bars depict mean ± SEM (*n* = 6). ***p < 0.001. **p <0.01. *p < 0.05. NS: Not significant. Student’s *t*-test was used for statistical analysis. **F** The average tumor weight was lower in the WT group than in the control and mutant (T174A) groups. Error bars depict mean ± SEM (*n* = 6). ***p < 0.001. *p < 0.05. NS: Not significant. Student’s *t*-test was used for statistical analysis.

Deletion of rodent *Lats1/2* completely abolishes phosphorylation of Yap at Ser112 (homologous to human YAP Ser127) and maintains high YAP activity, which is essential for transforming the properties of YAP-dependent cancers [13, 44]. In a colony forming assay, knockdown of *Yap/Taz* significantly reduced the colony-forming ability of MEF *Lats1/2* KO cells in soft agar (Fig. S7A), suggesting a decrease in oncogenic transforming activity [7, 13, 45]. We next established stable cell lines expressing TSSK1B WT or TSSK1B mutant (T174A) in MEF *Lats1/2* KO cells and performed soft agar colony-forming assays to assess the impact of TSSK1B on oncogenic transformation (Fig. S7B). Our finding revealed that TSSK1B WT significantly suppressed the colony-forming ability of MEF *Lats1/2* KO cells in soft agar, but not TSSK1B mutant (T174A), indicating that TSSK1B kinase activity is necessary for inhibiting the growth of MEF *Lats1/2* KO cells (Fig. 6A). However, TSSK1B could not suppress the colony-forming ability of MEF *Lats1/2* KO cells expressing HA–TEAD1ΔC–YAP(AD), which lacks Ser94 residue and exhibits highly constitutive activity (Fig. 6B). According to our model, TSSK1B may suppress anchorage-independent growth of MEF *Lats1/2* KO by phosphorylating Ser94 and inhibiting YAP. To validate our findings in vivo, we subcutaneously injected MEF *Lats1/2* KO cells stably expressing TSSK1B WT and TSSK1B mutant (T174A) cells into BALB/c nude mice (*N* = 6) and monitored tumor development. Within almost 4 weeks, tumors derived from TSSK1B WT cells were visibly smaller than those derived from TSSK1B mutant (T174A) cells (Figs. 6C and S7C). Time-course regression analyses demonstrated that TSSK1B WT induces slower tumor growth and considerably smaller tumor burden compared to the control and TSSK1B mutant (T174A), without significant toxicity or weight loss (Fig. 6D–F). These results are consistent with our in vitro findings and support the model that TSSK1B can inhibit YAP in the absence of *LATS1/2* in vivo. In conclusion, our findings indicate that TSSK1B inhibits the tumorigenic potential of YAP independently of LATS1/2 by phosphorylating YAP at Ser94, as demonstrated in in vitro colony forming assays and in vivo mouse models.

## Discussion

The phosphorylation of YAP is the key event in Hippo–YAP signaling and regulates the oncogenic transcriptional coactivators of YAP [4, 7, 46]. The Hippo pathway itself is regulated by various extracellular signals, such as physical contact, mechanical stimulation, nutrient intake, and hormonal factors [39, 47–50]. While the core upstream components of the Hippo pathway, including MST1/2, MAP4Ks, and LATS1/2 have been identified as key regulators of YAP/TAZ, it has been observed that their genetic ablation does not completely block YAP signaling, suggesting the existence of additional YAP regulators [38, 51, 52]. Recent studies have highlighted alternative pathways that can regulate YAP phosphorylation and inactivation independently of the core Hippo pathway components [3, 8, 46]. This study investigated the role of TSSK1B, a calcium/calmodulin-dependent protein kinase (CAMK) superfamily member, in regulating YAP and its impact on cellular proliferation and oncogenic transformation. Our findings demonstrate that TSSK1B negatively regulates YAP through two distinct pathways. Firstly, the LKB1–TSSK1B axis directly phosphorylates YAP at Ser94, inhibiting the formation of the YAP–TEAD complex and suppressing the expression of its target genes. The TSSK1B–LATS1/2 axis also phosphorylates YAP at Ser127, further contributing to its inhibition. Importantly, our study is the first to demonstrate that TSSK1B can phosphorylate YAP at Ser94, thus highlighting the role of TSSK1B in YAP regulation. The TSSK family exhibits unique expression patterns during spermatogenesis in the testis [24, 29]. Knockout (KO) mouse models have shown that Tssk1 and Tssk2 play roles in male infertility, affecting testis development and/or fertilization [53, 54]. While TSSK1B was initially identified as a testis-specific kinase, our study reveals its expression in other cell types, suggesting that its regulatory functions may extend beyond spermatogenesis. However, we acknowledge the limitation of primarily relying on cell culture models to analyze the interplay between The LKB1–TSSK1B axis and Hippo–YAP pathway in this study. While these models provide valuable insights, they may not fully recapitulate the intricate cellular environment present in living organisms. To address this concern, in vivo studies using animal models would provide a more comprehensive understanding of the physiological relevance of TSSK1B-mediated YAP regulation. This approach would not only strengthen the robustness of our findings but also open up new possibilities to explore the broader cellular context and potential applications of TSSK1B.

TSSK1B, located in the same branch of the AMPK kinase family on the human kinome tree, features a highly conserved T-loop threonine residue that undergoes phosphorylation and activation by an upstream kinase [31, 36, 55]. Distinguishing it from other AMPKs, TSSK1B functions as an auto-phosphorylating serine/threonine kinase [31, 32]. Considering their shared origin as members of the CAMK superfamily, we proposed that AMPK and TSSK1B might be regulated in a similar manner [31]. As expected, TSSK1B demonstrates a phosphorylation-dependent band shift in cells expressing LKB1 kinase, indicating direct phosphorylation of TSSK1B by LKB1 (Figs. 3C and S5C). Additionally, this phosphorylation event appears to be critical for determining the subcellular localization of TSSK1B (Figs. 3D, E). Investigations into spermatogenesis and sperm differentiation have explored the functions of both LKB1 and TSSK1B, although the substrates and in vivo mechanisms of action for TSSK1B remain largely unknown [24, 56]. Furthermore, recent studies have revealed the involvement of YAP in fine-tuning gene expression during fate commitment in spermatogenesis [57]. Our findings provide insights into the molecular mechanisms through which TSSK1B functions within the Hippo-YAP pathway and emphasize the significance of multilevel regulation in crucial cellular processes. Thus, investigating the cooperative relationship between LKB1, TSSK1B, and YAP phosphorylation can offer valuable insights into the complex mechanisms underlying spermatogenesis and fertility. By integrating these findings, we can gain a better understanding of the intricate processes involved in spermatogenesis and fertility.

The dysregulation of YAP activity has been strongly linked to tumorigenesis, positioning it as an attractive target for therapeutic interventions [18–20]. In light of our data, we have discovered that TSSK1B functions as an anti-tumor regulator by phosphorylating and inhibiting the oncogenic activity of YAP. This finding adds to our understanding of YAP regulation and expands the scope of YAP inhibition by TSSK1B. Importantly, TSSK1B exerts its inhibitory effects on YAP through both LATS1/2-dependent and -independent mechanisms, providing new insights into the intricate regulatory network of YAP. While our study identified TSSK1B’s impact on cellular proliferation and oncogenic transformation, further exploration is needed to gauge the clinical relevance of targeting TSSK1B for cancer therapy. Assessing the expression levels and activity of TSSK1B in cancer tissues becomes crucial in evaluating its therapeutic potential. Moreover, the broader implications of TSSK1B beyond its involvement in spermatogenesis underscore its relevance in diverse cellular contexts, necessitating ongoing investigation.

The implications of our study extend beyond fundamental research, as these findings hold promise for advancing our understanding of cellular signaling networks and informing the development of anticancer strategies. By targeting the dysregulated Hippo signaling pathway through interventions that leverage the regulatory role of TSSK1B, we may pave the way for future therapeutic approaches against cancer.

## Materials and methods

### Cell culture and transfection

A375P, HEK293A, HEK293T, MEF, and U2OS cells were cultured in DMEM containing 10% FBS, 50 units/mL of penicillin, and 50 μg/ml of streptomycin (Gibco). Cells were incubated in a humidified incubator with 5% CO_2_. Plasmid DNAs were transfected using a polyethylenimine reagent (PEI; Polysciences Inc.) according to the manufacturer’s instructions.

### Virus production and transduction

To produce retrovirus, HEK293T cells were transfected with retroviral vector, VSV-G, and GPE using PEI reagent. Lentivirus was produced by transfecting HEK293T cells with lentiviral vector, pMD2.G, and psPAX2 also using a PEI reagent. After incubation for 48 h, the retrovirus or lentivirus was filtered through a 0.45-µm PVDF filter and used to infect target cells with polybrene (Sigma-Aldrich). The transduced cells were incubated overnight at 37 °C, followed by replacement of the media after 15 h of infection. Appropriate antibiotic resistance markers were used to select the infected cells.

### Western blot and immunoprecipitation (IP)

Western blotting was performed using a standard protocol. For immunoprecipitation, cells were seeded and transfected with indicated plasmids. Cells were lysed using a mild lysis buffer (10 mM Tris, pH 7.5, 100 mM NaCl, 1 mM Na_3_VO_4_, 1 mM EDTA, 100 mM NaCl, 1% NP-40, 50 mM NaF, and protease inhibitor cocktail) and centrifuged at 13,000 rpm for 15 min at 4 °C. The resulting supernatants were subjected to immunoprecipitation using appropriate antibodies at 4 °C for 2 h. Protein A/G magnetic beads (Thermo Fisher Scientific Inc.) were then added to the immunoprecipitated complexes and incubated for 1 h at 4 °C. The immunoprecipitated complexes were washed with the mild lysis buffer and boiled with Laemmli buffer for SDS-PAGE. The antibodies used are listed in Supplementary Table S1.

### Subcellular fractionation

Cells were lysed with a buffer A (10 mM HEPES, 1.5 mM MgCl_2_, 10 mM KCl, 0.5 mM DTT, and 0.05% NP-40 pH7.9) containing with protease inhibitors for 5 min and centrifuged at 3000 rpm at 4 °C. The supernatants were used for cytoplasmic samples, and the pellets were resuspended in buffer B (5 mM HEPES, 1.5 mM MgCl2, 0.2 mM EDTA, 0.5 mM DTT, 26% glycerol pH 7.9). Subsequently, the pellets were sonicated for 10 sec to break the plasma membrane. After sonication, the lysed cells were incubated at 4 °C for 30 min and then centrifuged at 13,000 rpm for 10 min. The supernatants were used with nuclear samples.

### Luciferase assay

Cells were plated onto 12-well plates and transfected with Gal4-TEAD4, 5хUAS-luc reporter, Renilla, and the indicated plasmids. After 48 h of incubation, cells were lysed and luciferase activity was measured using a Dual-Glo® luciferase reporter assay kit (Promega) according to the manufacturer’s protocol. Luminescence activity was normalized to the activity of Renilla luciferase activity.

### Recombinant protein purification

The indicated genes were cloned into pGEX-KG vector, which was transformed into *E. coli* BL21 cells. The recombinant GST-fusion proteins were induced by addition of 0.5 mM IPTG, followed by overnight growth at 18 °C. The supernatant was discarded and the cell pellets were lysed in PBS containing 1% Triton X-100, 1 mM PMSF, 5 mM β-mercaptoethanol, and 2 mM EDTA using ultrasonication. After centrifugation, the supernatant was incubated with GSH beads for 1 h at 4 °C, washed with PBS, and then washed with 20 mM Tris-HCl (pH 8.0). The bound proteins were eluted with elution buffer containing 20 mM glutathione, and 50 mM Tris-HCl (pH 8.0) in distilled water.

### In vitro kinase assay

Cells were lysed with a lysis buffer containing protease inhibitor cocktail (Roche), and anti-indicated antibodies were used for immunoprecipitation. Immunoprecipitated kinases were washed with mild lysis buffer thrice and then subjected to a kinase assay using purified GST-YAP or GST-TSSK1B as substrates. The reactants were terminated with Laemmli buffer after 1 h of incubation at 30 °C. Phosphorylation of substrates were determined by phospho-Abs or and ^32^P-autoradiography.

### Immunofluorescence staining

Cells were cultured on coverslips, fixed with 2% formaldehyde in PBS for 15 min and permeabilized with 0.2% Triton X-100 in PBS for 5 min. After blocking with 10% FBS in PBS, cells were incubated with primary Abs in blocking buffer overnight at 4 °C. Subsequently, cells were washed with PBS, incubated with Alexa Flour-conjugated secondary Abs for 1.5 h, and washed three times with PBS. To stain the nucleus, cells were counterstained with 4’,6-diamidino-2-phenylindole (DAPI). The coverslips were mounted with Gel/Mount solution (Biomeda) and images were captured using a confocal microscope (LSM980 NLO) at the Three-Dimensional Immune System Imaging Core Facility of Ajou University. The ZEN 3.5 blue edition program was used for data analysis.

### RNA isolation and quantitative real-time polymerase chain reaction (qRT-PCR)

RNA was isolated from cells or mouse tissues by utilizing TRIzol reagent (Invitrogen). cDNA was synthesized from 1 µg of total RNA using GoScript reverse transcriptase (Promega), followed by qRT-PCR using a KAPA SYBR FAST qPCR Master Mix (2×) kit (KAPA Biosystem) according to the manufacturer’s instructions and StepOnePlus™ Real-Time PCR system (Thermo Fisher Scientific Inc.). Normalization to hypoxanthine-guanine phosphoribosyltransferase 1 (*HPRT1*) mRNA was utilized to calculate the relative abundance of mRNA. The primer sequences are provided in the Supplementary Table S1.

### Site-directed mutagenesis

For the mutagenic primers, we used New England Biolabs (NEB) primer design software, NEBaseChanger. Flag-TSSK1B mutant constructs were generated using a Q5 Site-Directed Mutagenesis Kit (NEB) according to the manufacturer’s protocol. The primers used in site-directed mutagenesis are described in the Supplementary Table S1.

### Generation of knockout cell lines using CRISPR/Cas9 system

To generate Knockout (KO) cell lines, single guide RNAs (sgRNAs) were designed using http://rgenome.net/cas-designer/ and cloned into Lenti-CRISPR v2 vector (Addgene plasmid #52961). The resulting constructs were transfected into HEK293T cells to produce lentivirus that was used to infect the target cells. The transduced cells were then selected using puromycin and sorted by FACS into 96-well plates to isolate single colonies. The knockout of targeted genes in the single clones was confirmed by immunoblotting and validated (CRISPR induced genetic changes) by sequencing using TA cloning kit. Details of the sgRNA sequences are provided in Supplementary Table S1.

### TA cloning

TA cloning experiment was performed using the AccRapid™ cloning kit (Bioneer) to obtain the insert DNA from genomic DNA extracted from the knockout cell line using the AccuPrep® Genomic DNA extraction kit (Bioneer). Taq polymerase (PCRBIO) was used for PCR amplification. The PCR product was purified using the FavorPrep GEL/PCR Purification Mini kit (FAVORGEN) and ligated with pBHA-T vector using T4 ligase at 16 °C overnight. The recombinant plasmids were transformed into competent cells, and white colonies were selected on Luria-bertani (LB) plates containing IPTG and X-Gal after incubation at 37 °C. The inserts were identified using the basic local alignment search tool available in the National Center for Biotechnology Information (NCBI).

### siRNA transfection

The ON-TARGETplus siRNA targeting human YAP, human WWTR1, mouse Yap, and mouse Wwtr1 were purchased from Dharmacon. On-TARGETplus Non-targeting Control pool was used as a negative control, and the transfection of siRNA carried out using RNA iMAX (Invitrogen), following manufacturer’s protocol. The information of siRNA is described in Supplementary Table S1.

### shRNA transduction

The shRNAs targeting TSSK1B (TRCN0000219667 and TRCN0000219668) and the control shRNA (pLKO.1) were purchased from Merck. The lentiviral particles were generated using pMD2.G and psPAX2 in HEK293T cells.

### Sulforhodamine B (SRB) assay

Cells were seeded in 96-well plates and incubated at 37 °C for 18 h. The plates were then harvested every two days and cells were fixed with 10% trichloroacetic acid solution (Sigma-Aldrich) at 4 °C overnight. After being washed thrice with distilled water, the cells were stained with 0.4% SRB in a 1% acetic acid solution in darkness for 30 min. The SRB-stained cells were dissolved in 10 mM Trizma base (Sigma-Aldrich) and incubated on an orbital shaker for 1 h. Finally, the resolved SRB-stained cells were quantified using a microplate spectrophotometer (BioTek Instruments) at 540 nm wavelength.

### Soft agar assay

For the soft agar assay, the bottom layer of each ultra-attachment 6-well plate was coated with 0.7% agar in 2 ml of DMEM containing 10% FBS. Cells were suspended in 1.5 ml of top layer with 0.35% agar in DMEM containing 10% FBS and added to each well. The cells were incubated for 3 weeks, with fresh medium replaced every three days. The colonies were stained with 0.05% crystal violet, photographed using a phase-contrast microscope (Olympus 1 × 71 DP controller) at 200X magnification. A scale bar of 200 µm was included in the images. The quantification of colonies was determined by counting the number of colonies that were equal to or larger than 200 µm in each well and calculating the average.

### Xenograft

BALB/c nude mice were purchased from Orientbio Inc. (Shizuoka). Xenograft experiments were conducted in accordance with the institutional procedure of Laboratory Animal Research Center of Ajou University Medical Center. A total of 1.5 × 10^6^ cells was injected into the subcutaneous space on the back of 8-week-old male nude mice. The body and tumor weights of the mice were monitored and recorded for a period of 4 weeks, after which the mice were euthanized for further analysis.

### Statistical analysis

Statistical analysis was performed using GraphPad Prism software version 8.0. Results are shown as mean ± standard error of the mean (S.E.M.). Statistical analysis was carried out using Student’s t-test (unpaired, two-tailed) or two-way ANOVA, with *p < 0.05, **p < 0.01, and ***p < 0.001 indicating statistical significance. No statistical method was used to determine sample size. The investigators were blinded to allocation during experiments and outcome assessment.

## Supplementary information

Supplemental figures, Supplemental legend, Supplemental table

Original Data File

## Data Availability

All data generated and/or analysed during this study are available from the corresponding author on reasonable request.
