# Correction: The LKB1–TSSK1B axis controls YAP phosphorylation to regulate the Hippo–YAP pathway

**DOI:** 10.1038/s41419-024-07238-9

**Published:** 2024-11-26

**Authors:** Cho-Long Kim, Su-Bin Lim, Sue-Hee Choi, Dong Hyun Kim, Ye Eun Sim, Eun-Hye Jo, Keeeun Kim, Keesook Lee, Hee-Sae Park, Su Bin Lim, Li-Jung Kang, Han-Sol Jeong, Youngsoo Lee, Carsten G. Hansen, Jung-Soon Mo

**Affiliations:** 1 Department of Biomedical Sciences, Graduate School, Ajou University School of Medicine, Suwon, 16499 South Korea; 2 School of Biological Sciences and Technology, Chonnam National University, Gwangju, 61186 South Korea; 3 Institute of Medical Science, Ajou University School of Medicine, Suwon, 16499 South Korea; 4 Department of Biochemistry and Molecular Biology, Ajou University School of Medicine, Suwon, 16499 South Korea; 5 Three-Dimensional Immune System Imaging Core Facility, Ajou University, Suwon, 16499 South Korea; 6 Division of Applied Medicine, School of Korean Medicine, Pusan National University, Yangsan, 50612 South Korea; 7 The University of Edinburgh, Institute for Regeneration and Repair, Centre for Inflammation Research, Edinburgh BioQuarter, Edinburgh, UK

**Keywords:** Cell growth, Phosphorylation, Oncogenes

Correction to: *Cell Death & Disease* 10.1038/s41419-024-06465-4, published online 20 January 2024

In this article, the funding note has been corrected. It should read: This work was supported by the National Research Foundation of Korea (NRF) Grants NRF-2020R1A2C2011016 (J-SM), and 2021R1I1A1A01051938 (L-JK) and the Korea Basic Science Institute (National Research Facilities & Equipment Center) grant No. 2019R1A6C1010003. The Hansen lab is supported by ISSF3 and Worldwide Cancer Research. This work was supported by research fund of Ajou University Medical Center (2023).

The original article has been corrected.

