# Supplementary material for: The LKB1–TSSK1B axis controls YAP phosphorylation to regulate the Hippo–YAP pathway
Source: Cell Death Dis. 2024 Jan 20;15(1):76. doi: 10.1038/s41419-024-06465-4 (PMC10799855; doi:10.1038/s41419-024-06465-4)
Supplement: Supplementary file 1 — Supplemental figures, Supplemental legend, Supplemental table [file 41419_2024_6465_MOESM1_ESM.pdf]

Supplementary Figure 1

A

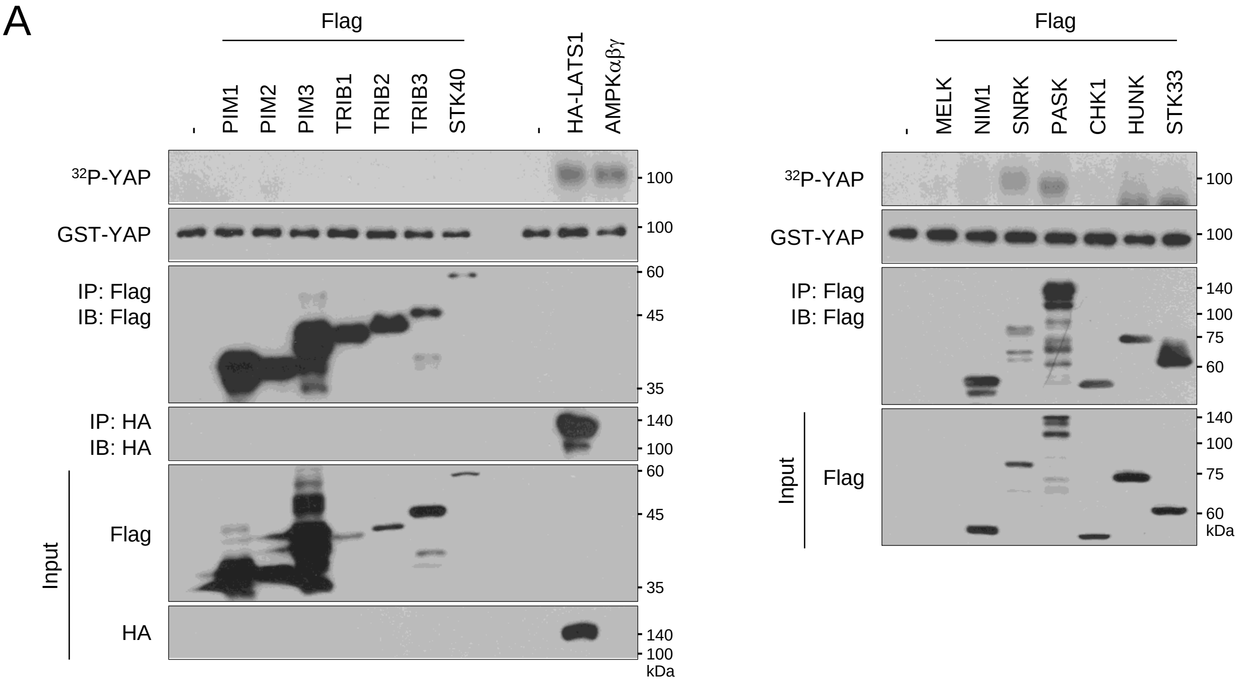

B

```
Homo sapience
AMPKα1  148   RDLKPENVLLDA-HMNAKIADFGLSNMMSD----------------GEFLRTSCGSPNYAAPEVISGRLYAGPEVDIWSSG
AMPKα2  137   RDLKPENVLLDA-HMNAKIADFGLSNMMSD----------------GEFLRTSCGSPNYAAPEVISGRLYAGPEVDIWSCG
TSSK1B  134   RDLKCDNLLLDK-DFNIKLSDFSFSKRCLRD-----------DSGRMALSKTFCGSPAYAAPEVLQGIPYQPKVYDIWSLG
TSSK2   134   RDLKCENLLLDK-DFNIKLSDFGFSKRCLRD-----------SNGRIILSKTFCGSAAYAAPEVLQSIPYQPKVYDIWSLG
TSSK3   132   RDLKCENALLQG--FNLKLTDFGFAKVLP--------------KSHRELSQTFCGSTAYAAPEVLQGIPHDSKKGDVWSMG
TSSK4   156   RDLKLENLLLDK-WENVKISDFGFAKMVPSNQPVGCSPSYRQVNCFSHLSQTYCGSFAYACPEILRGLPYNPFLSDTWSMG
TSSK6   133   RDLKCENVLLSPDERRVKLTDFGFGRQA---------------HGYPDLSTTYCGSAAYASPEVLLGIPYDPKKYDVWSMG
              ****  * **       *  **                             * ***  ** **           * ** *

Mus musculus
Ampkα1  148   RDLKPENVLLDAH-MNAKIADFGLSNMM----------------SDGEFLRTSCGSPNYAAPEVISGRLYAGPEVDIWSSG
Ampkα2  137   RDLKPENVLLDAQ-MNAKIADFGLSNMM----------------SDGEFLRTSCGSPNYAAPEVISGRLYAGPEVDIWSSG
Tssk1   134   RDLKCENLLLDKD-FNIKLSDFGFAKVLPK--------------SRRELSQTFCGSTAYAAPEVLQGIPHDSKKGDVWSMG
Tssk2   134   RDLKCENLLLDKD-FNIKLSDFGFSKRCLRDD-----------SGRLILSKTFCGSAAYAAPEVLQGIPYQPKVYDIWSLG
Tssk3   132   RDLKCENALL-QG-FNLKLTDFGFGRQA---------------HGYPDLSTTYCGSAAYASPEVLLGIPYDPKKYDVWSLG
Tssk4   156   RDLKLENLLLDKR-ENVKISDFGLSNMM----------------SDGEFLRTSCGSPNYAAPEVISGRLYAGPEVDIWSCG
Tssk6   133   RDLKCENVLLSPDERRVKITDFGFAKMVPSSQPVHSSPSYRQMNSLSHLSQTYCGSFAYACPEILLGLPYNPFLSDTWSMG
              **** ** **       *  ***                            * ***  ** **   *       * ** *

Drosophila melanogaster
AMPKα sub 180   LLLDHNMHVKIADFGLSNMMLDGE-----FLRTSCGSPNYAAPEVISGKLYAGPEVD
Tssk1     184   ILLSKRLNVKLADFGFARYC-RDESGHQLKSETYCGSAAYAAPEVVCGVPYDPKMAD
                 **      *  **                  * ***  ******  *        *
```

Supplementary Figure 2

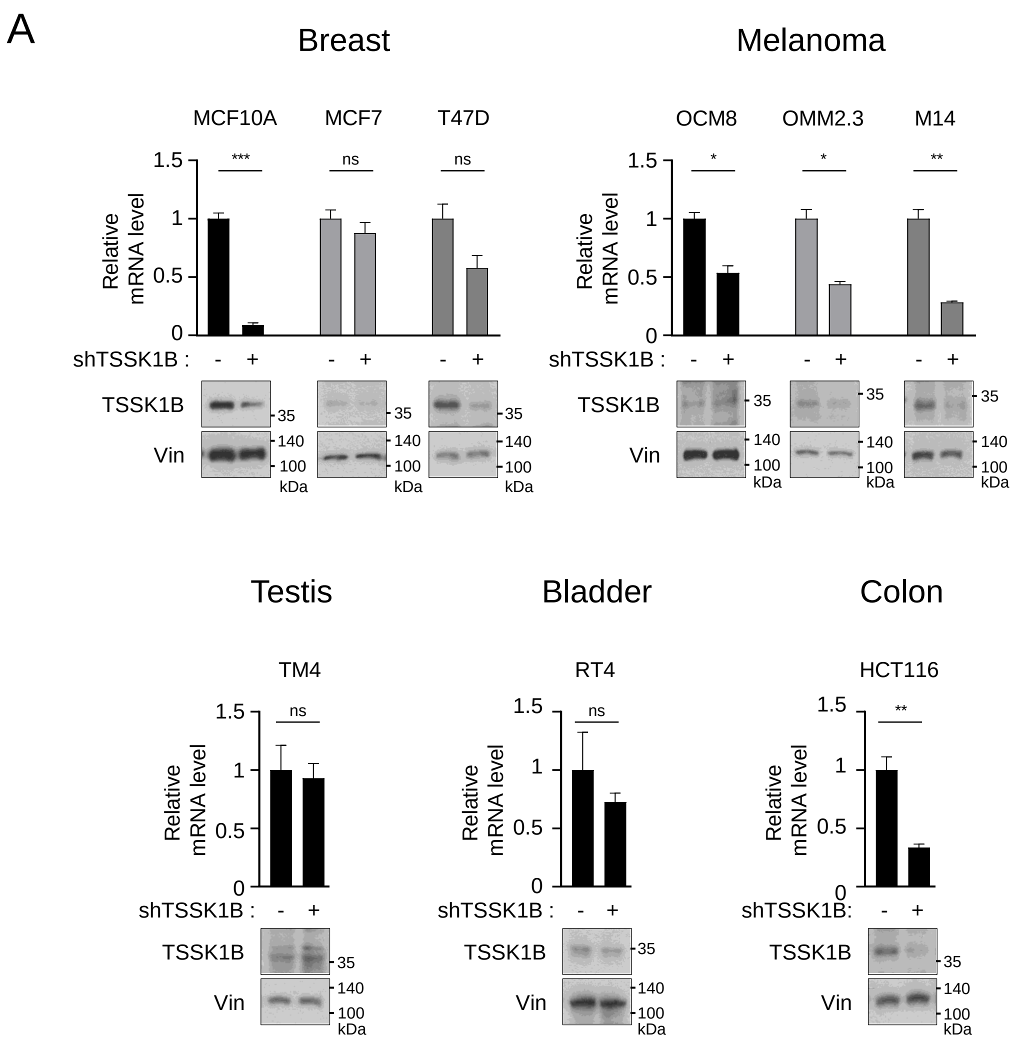

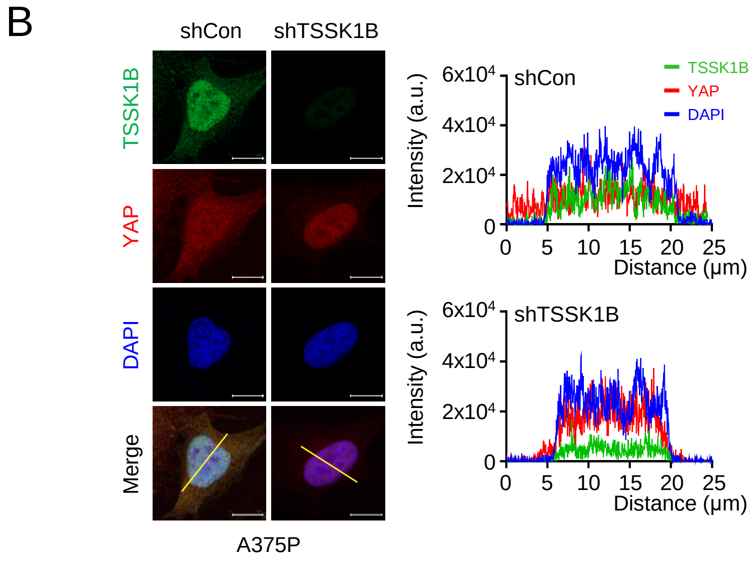

Supplementary Figure 3

A

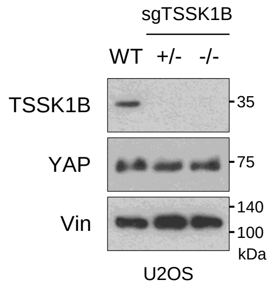

B

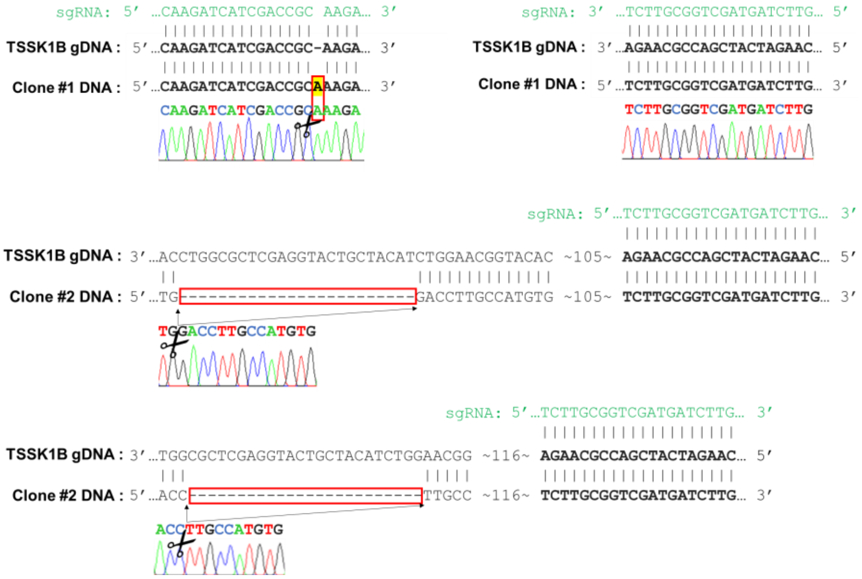

Supplementary Figure 4

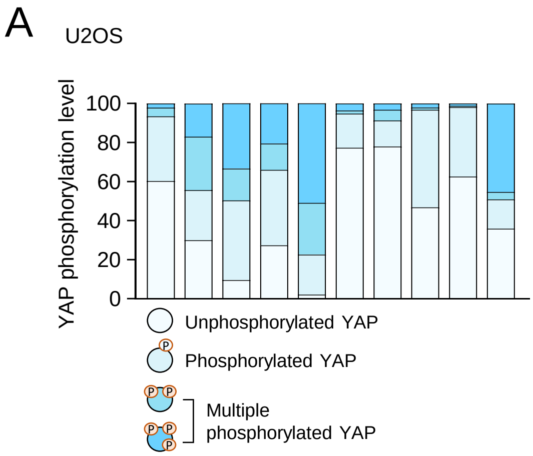

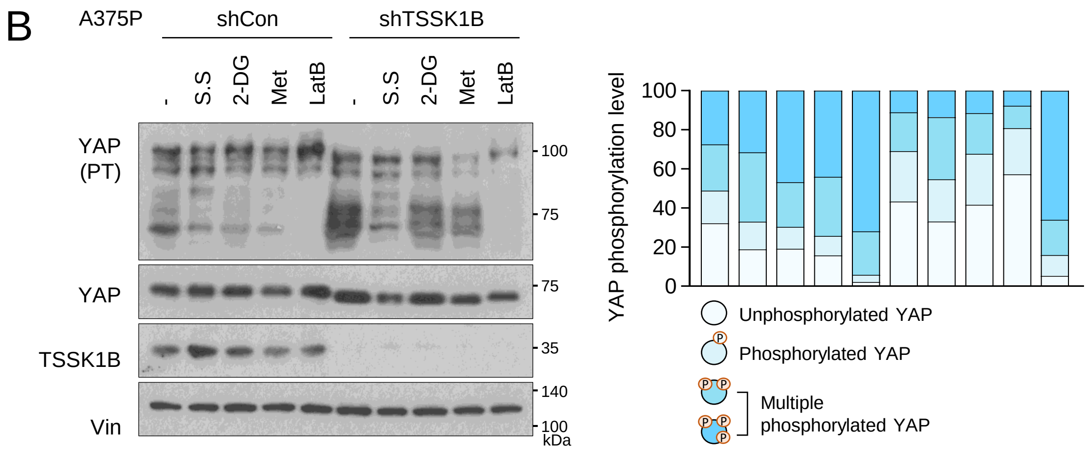

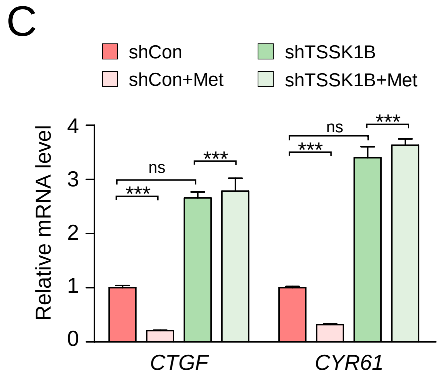

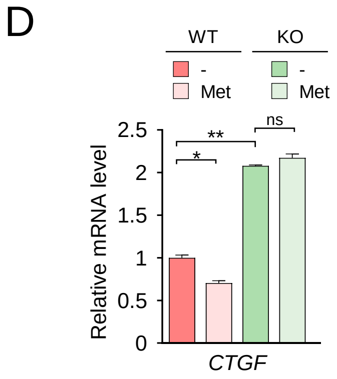

Supplementary Figure 5

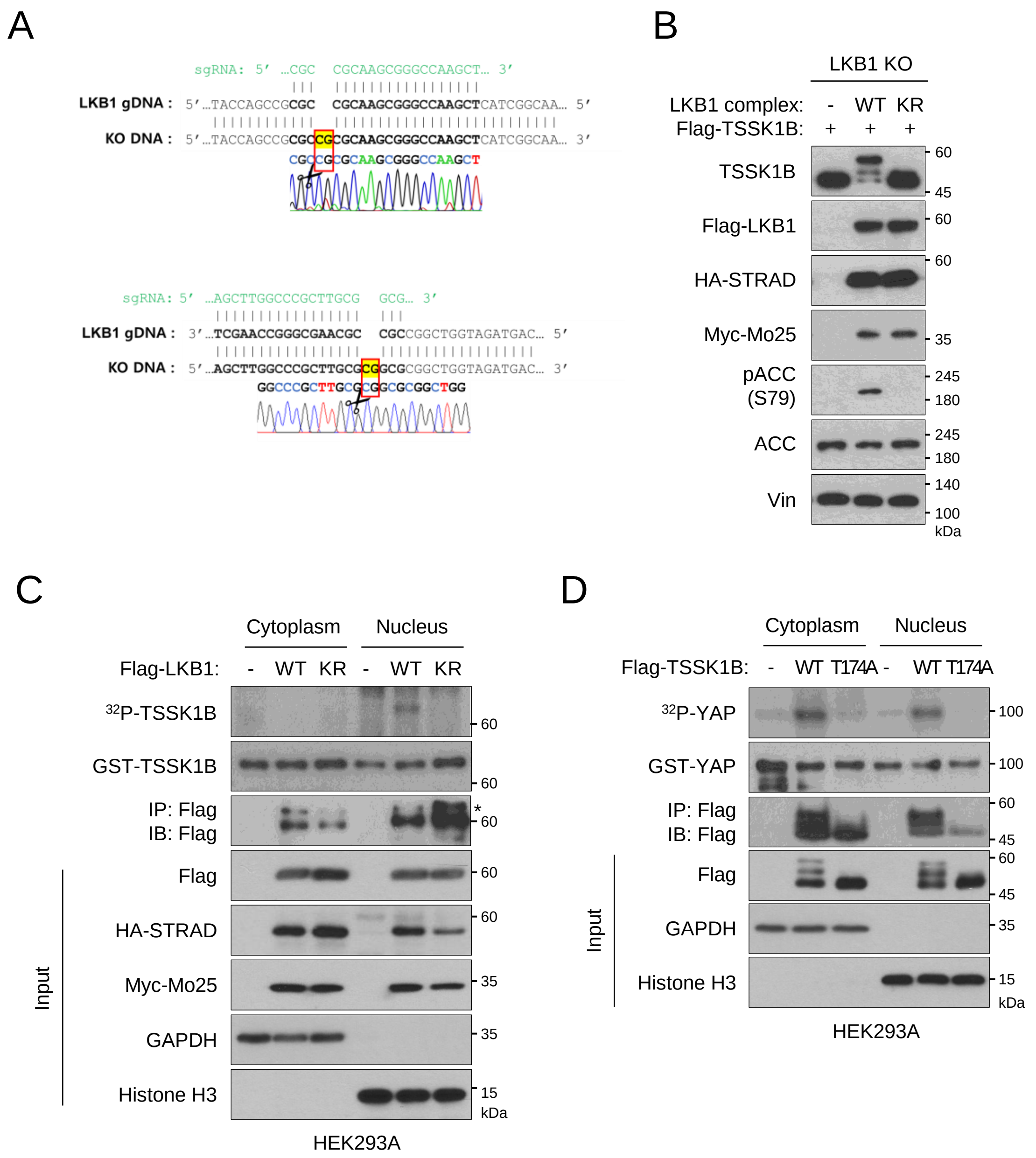

Supplementary Figure 6

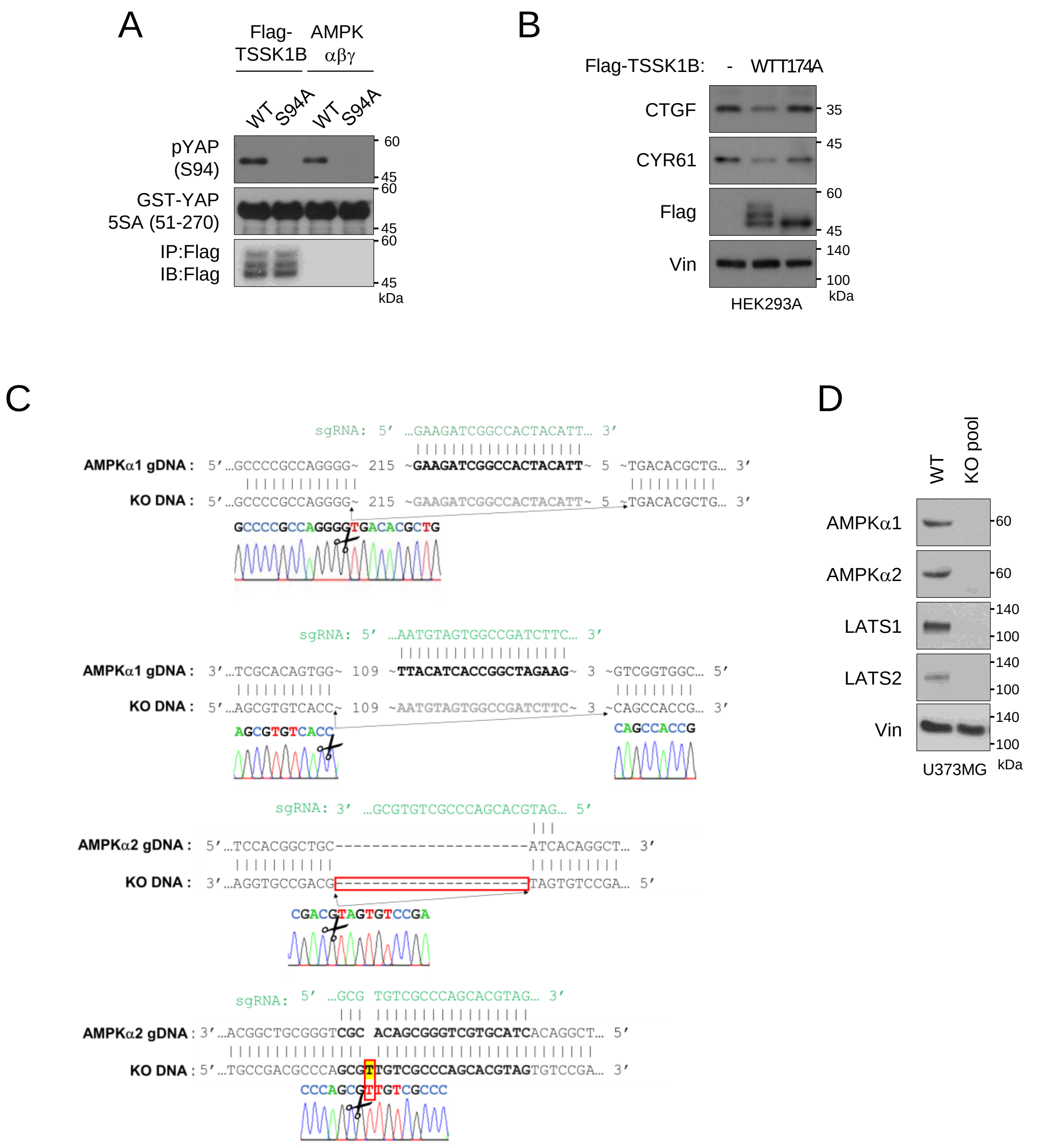

Supplementary Figure 7

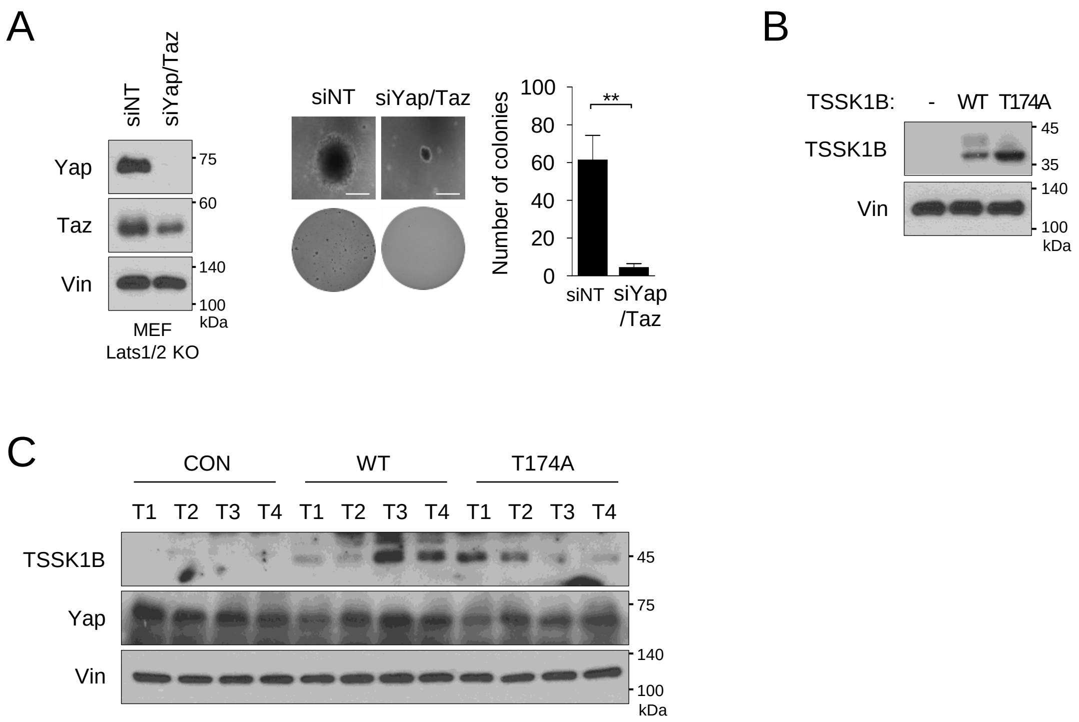

Supplementary Figures

Supplementary Figure 1, related to Figure 1

***In vitro* screening of the CAMK family for YAP kinases**

(A) Flag-tagged individual kinases, including those of the CAMK family, and HA-LATS1 were expressed in HEK293A cells and immunoprecipitated using anti-Flag or anti-HA Abs. Immunoprecipitated kinases and purified AMPKαβγ complexes were used for the kinase reaction with GST-YAP. Phospho-YAP was analyzed using ^{32}P autoradiography, and the total amount of GST-YAP was detected by immunoblotting using anti-GST Ab. (B) Sequence alignment showing the conservation of the T-loop sequence in TSSKs and AMPK kinase family across different species. Residues in red are conserved amino acids among the TSSK family and AMPK1/2; the critical Threonine residue is highlighted in yellow.

Supplementary Figure 2, related to Figure 2

Knockdown of T*SSK1B* in various cell lines

(A) Cell lines were treated with shCon and shTSSK1B and qRT-PCR was performed using specific primer pairs to quantify the mRNA levels of *TSSK1B*. The measurements were normalized to the *HPRT1* mRNA levels. Error bars depict mean ± SEM (n = 3). ***$p < 0.001$. **$p < 0.01$. *$p < 0.05$. NS: Not significant. Student's *t*-test was used for statistical analysis. Lysates from tumor cell lines were transduced using shCon or shTSSK1B were subjected to immunoblotting with antibody to human TSSK1B. (B) HEK293A cells treated with shCon or shTSSK1B were subjected to immunofluorescence staining for TSSK1B (green) and YAP (red); 4',6-diamidino-2-phenylindole (DAPI) (blue) was used for nuclear staining. Scale bars, 10 μm. The fluorescence intensity of TSSK1B, YAP, and DAPI was quantified by drawing a line (yellow) of 25 μm in a merged figure using the ZEN 3.5 blue edition program.

Supplementary Figure 3, related to Figure 2

Characterization of *TSSK1B* knockout in U2OS cells

(A) Knockout of *TSSK1B* gene in U2OS cells. Whole cell lysates were subjected to immunoblotting. (B) Sequencing analysis of CRISPR-mediated *TSSK1B* KO in U2OS cells. A schematic graph shows the genomic sequencing results before and after the paired target single guide RNA (sgRNA)-mediated insertion or deletion. The red boxes represent the starting point from which CRISPR/Cas9 system operates. Sanger sequencing was used to analyze the correlation between DNA fragments.

Supplementary Figure 4, related to Figure 2

TSSK1B is required for stress-induced phosphorylation of YAP, leading to its inhibition of transcriptional activity

(A) The relative ratio of phosphorylated YAP to unphosphorylated YAP was detected using a phos-tag gel and analyzed with the AlphaEaseFC program. The bar graphs depict the ratio of each band to YAP on the phos-tag gel. (B) A375P cells treated with shCon and shTSSK1B were incubated with serum-free medium (S.S) for 2 h, 25 mM 2-Deoxy glucose (2-DG) for 2 h, 1 mM metformin (Met) for 4 h, or 0.25 $\mu g \cdot ml^{-1}$ latrunculin B (LatB) for 30 min, and whole cell lysates were immunoblotted. The bar graphs depict the ratio of each band to YAP on the phos-tag gel. (C) shCon- or shTSSK1B-treated A375P cells were incubated with 1 mM metformin for 4 h. The mRNA levels of *CTGF* and *CYR61* were determined. The relative unit values for mRNA were normalized to one with respect to that of the control group without metformin treatment. Error bars depict mean ± SEM (n = 3). ***$p < 0.001$. NS: Not significant. Student's *t*-test was used for statistical analysis. (D) U2OS WT and *TSSK1B* KO cells were treated with 1 mM metformin for 4 h. The mRNA levels of *CTGF* were determined using qRT-PCR. The relative unit values for mRNA were normalized to one with respect to that of the control group without metformin treatment. Error bars depict mean ± SEM (n = 3). **$p < 0.01$. *$p < 0.05$. NS: Not significant. Student's *t*-test was used for statistical analysis.

Supplementary Figure 5, related to Figure 3

LKB1 is critical for the phosphorylation of TSSK1B

(A) The sequencing analysis of *LKB1* KO is presented through a schematic graph that shows the genomic sequencing

results after sgRNA-mediated *LKB1* deletion in HEK293A cells. (B) HEK293A *LKB1* KO cells were co-transfected with Flag-TSSK1B together with Flag-LKB1 WT or KR with HA-STRAD and Myc-Mo25 constructs. Cell lysates were subjected to immunoblotting using the indicated Abs. (C) Flag-LKB1 WT or KR, HA-STRAD, and Myc-Mo25 were expressed in HEK293A cells and fractionated into nuclear and cytoplasmic fractions. Nuclear and cytoplasmic fractions were immunoprecipitated with anti-Flag, and immunoprecipitated LKB1 kinases were used for the kinase reaction with GST-TSSK1B. pTSSK1B was analyzed using ^{32}P autoradiography, and GST-TSSK1B was detected by immunoblotting. (D) Flag-TSSK1B WT or mutant (T174A) was expressed in HEK293A cells and fractionated into nuclear and cytoplasmic fractions. Nuclear and cytoplasmic fractions were immunoprecipitated with anti-Flag, and immunoprecipitated TSSK1B kinases were used for the kinase reaction with GST-YAP. pYAP was analyzed using ^{32}P autoradiography, and GST-YAP was detected by immunoblotting. (C-D) Histone H3 and GAPDH were markers for nuclear (N) and cytoplasmic (C) fractions, respectively.

Supplementary Figure 6, related to Figure 5

TSSK1B-mediated phosphorylation of YAP at Ser94

(A) Flag-TSSK1B was expressed in HEK293A cells and immunoprecipitated using anti-Flag Ab. Immunoprecipitated Flag-TSSK1B and purified AMPKαβγ complexes were used for the kinase reaction with the substrate GST-YAP 5SA fragment (amino acid residues 51–270). The phosphorylation levels of YAP were detected by immunoblotting using anti-pYAP Ser94 Ab, and the total amount of GST-YAP was detected by immunoblotting using anti-GST Ab. The GST-YAP (S94A) mutant was used as a negative control as a non-phosphorylated substrate in the kinase reaction. (B) Cell lysates from HEK293A cells expressing Flag-TSSK1B WT or mutant (T174A) were subjected to immunoblotting. (C) The sequencing analysis of *AMPKα1* and *AMPKα2* was presented through a schematic graph that showed the genomic sequencing results after the target gRNA-mediated deletion of *AMPKα1/2*. (D) Knockout of *AMPKα1/2–LATS1/2* genes in parental U373MG cell pools. Protein levels of AMPKα1/2 and LATS1/2 were detected to immunoblotting using the indicated Abs.

Supplementary Figure 7, related to Figure 6

Knockdown of *Yap/Taz* suppresses anchorage-independent growth in MEF *Lats1/2* KO cells

(A) Knockdown of *Yap/Taz* suppresses anchorage-independent growth in MEF *Lats1/2* KO cells. MEF *Lats1/2* KO cells were transfected using siRNA targeting Yap/Taz, and knockdown efficiency of *Yap/Taz* was confirmed using the indicated Abs. *Yap/Taz* deficient cells were assessed for colony formation in soft agar. Error bars depict mean ± SEM (n = 3). $^{**}p < 0.01$. Student's t-test was used for statistical analysis. Scale bars, 200 µm. (B) pBABE-vector (-), pBABE-TSSK1B WT or mutant (T174A) were stably expressed in MEF *Lats1/2* KO cells and whole cell lysates were subjected to immunoblotting using the indicated Abs. (C) TSSK1B expression in mouse tumor. The mouse tumor tissues were lysed and the expression of TSSK1B WT and mutant (T174A) were detected by immunoblotting using anti-TSSK1B and anti-Yap Abs. T: Tumor tissues

Supplementary Table

REAGENT	*SOURCE*	*IDENTIFIER*
**Antibodies**		
Acetyl-CoA Carboxylase (C83B10) Rabbit	Cell Signaling Technology	3676
Alexa Fluor 488-conjugated goat anti-mouse IgG (H+L)	Invitrogen	A11029
Alexa Fluor 594-conjugated goat anti-rabbit IgG (H+L)	Invitrogen	A11037
AMPKα Rabbit	Cell Signaling Technology	2532
AMPKα2 Rabbit	Cell Signaling Technology	2757
ANTI-FLAG® M2-Peroxidase (HRP) Mouse	Sigma-Aldrich	A8592
Anti-Histone H3 antibody	Abcam	Ab1791
CTGF (L-20) Goat	Santa Cruz Biotechnology Inc	sc-14939
Cyr61 (H-78) Rabbit	Santa Cruz Biotechnology Inc	sc-13100
FLAG® M2 Mouse	Sigma-Aldrich	F1804
GAPDH Antibody	Avivasysbio	OAEA00006
GFP (B-2) Mouse	Santa Cruz Biotechnology Inc	sc-9996
GST (56C1) Mouse	Santa Cruz Biotechnology Inc	sc-80998
HA-probe (12CA5) Mouse	Santa Cruz Biotechnology Inc	sc-57592
HGK Rabbit	Cell Signaling Technology	3485
LATS1 (C66B5) Rabbit	Cell Signaling Technology	3477
LATS2 (D83D6) Rabbit	Cell Signaling Technology	5888
LKB1 (D60C5) Rabbit	Cell Signaling Technology	3047
Monoclonal Anti-Vinculin antibody	Sigma-Aldrich	V9131
Mouse IgG HRP Linked Whole Ab	Cytiva	NA931
MST2 Rabbit	Cell Signaling Technology	3952
Myc (9E10) Mouse	Santa Cruz Biotechnology Inc	sc-40
Phospho-Acetyl-CoA Carboxylase (Ser79) Rabbit	Cell Signaling Technology	3661
Phospho-LATS1 (Thr1079) Rabbit	Cell Signaling Technology	9159
Phospho-YAP (Ser127) Rabbit	Cell Signaling Technology	4911
Purified Mouse Anti-Human MST1	BD Biosciences	611052
Rabbit IgG HRP Linked Whole Ab	Cytiva	NA934
TSSK1B (NQ-A36) Mouse	Santa Cruz Biotechnology Inc	sc-135590
YAP (D8H1X) XP® Rabbit	Cell Signaling Technology	14074
YAP/TAZ (D24E4) Rabbit	Cell Signaling Technology	8418
YAP1 (63.7) Mouse	Santa Cruz Biotechnology Inc	sc-101199
YAP1 (Ser94) Rabbit	Abbiotec	254542
**Chemicals**		
1x RBC lysis buffer	Invitrogen	501129751

2-deoxy-D-glucose (2-DG)	Sigma-Aldrich	D8375
6well culture plate, surface Treatment, sterile	SPL	30006
12well Cell Culture Plate, Surface Treatment, Sterile	SPL	30012
90mm Cell Culture Dish (90x20), Surface Treatment	SPL	20101
96well Cell Culture Plate, Surface Treatment, Sterile	SPL	30096
2-Mercaptoethaol	Sigma-Aldrich	M3148
ATP, [γ-^{32}P]- 3000Ci/mmol 10mCi/ml EasyTide Lead, 250 µCi	PerkinElmer	NEG502A250UC
Bovine Serum Albumin (BSA)	RMBIO	BSA-BSH
Collagenase A	Sigma-Aldrich	10103578001
Crystal Violet	Junsei	27200-1210
Costar® Ultra-Low Attachment Multiple Well Plate	Corning	CLS3471
4’,6-diamidino-2-phenylinodole (DAPI)	Tocris	5748
Difco™ Agar Noble	BD	214220
DMEM, powder, high glucose, pyruvate	Gibco	12800082
DMEM/F-12, powder	Gibco	12500062
DMEM/F-12, 1:1 Mixture (1X) with phenol red, liquid	Welgene	LM002-04
DMEM/F-12, 1:1 Mixture (1X) without phenol red, liquid	Welgene	LM002-05
DNase I	Sigma-Aldrich	10104159001
dNTP mixture (2.5mM)	Takara	4030
Dithiothreitol (DTT)	Goldbio	DTT10
Ethylenediaminetetraacetic acid (EDTA)`	Sigma-Aldrich	E26282
Formaldehyde solution	Sigma-Aldrich	F8775
Gel/mount	Biomeda corporation	M01
Gibco® Certified FBS	Gibco	16000-044
Glycerol	Samchun	G0272
HEPES	VWR	VWRC0511
Immobilon-P PVDF	Millipore	IPVH00010
Immobilon Western Chemiluminescent HRP substrate	Millipore	WBKLS0500
Isopropyl-beta-D-thiogalactoside (IPTG)	Goldbio	I2481
Latrunculin B (LatB)	Sigma-Aldrich	L5288
Magnesium chloride hexa-hydrate	Sigma-Aldrich	M9272
Metformin hydrochloride (Met)	Tocris Bioscience	2864
Nonidet P40 (NP40)	Sigma-Aldrich	74385
DPBS, powder, no calcium, no magnesium	Gibco	21600-010
Paraformaldehyde	Sigma-Aldrich	158127
Penicillin-streptomycin (10,000U/mL)	Gibco	15140122
Phos-tag™ Acrylamide	Wako Pure Chemical Industries Ltd	304-93521

Polybrene Infection	Sigma-Aldrich	TR-1003-G
Polyethylenimine (PEI) reagent	Polysciences Inc	24885
Potassium chloride	Sigma-Aldrich	746436
Phenylmethylsulfonyl fluoride (PMSF)	Goldbio	P-470
PrimeSTAR® Max DNA Polymerase	Takara	R045A
Protease inhibitor cocktail	Sigma-Aldrich	P8340
Pierce™ Protein A/G magnetic Beads	Thermo Fisher	88802
Random primer (9mer)	Takara	3802
Recombinant AMPKαβγ complex	GenScript	Z02516
Recombinant RNase inhibitor	Takara	2313A
Reverse transcriptase	Promega	A5004
RNAiMAX	Invitrogen	10601435
RPMI Medium 1640	Gibco	31800-014
Sodium bicarbonate	Sigma-Aldrich	S5761
Sodium chloride	Samchun	S2097
Sulforhodamine B (SRB)	Sigma-Aldrich	230162
Syringe filter 0.45µm	Satorius	16555K
PCRBIO Taq DNA polymerase	PCRBIO	PB10.11
Trichloroacetic acid solution	Sigma-Aldrich	T0699
Trizma base	Sigma-Aldrich	77-86-1
TRIzol™ reagent	Invitrogen	15596018
Triton X-100, Reagent Grade	Amresco	0694-1L
Trypsin/EDTA 500ml	Gibco	TFS-25200072
Water, Ultra pure	Biosesang	WR2006-100-00
X-Gal	Enzynomics	XGAL1000
**Assays**		
AccuPrep® Genomic DNA Extraction Kit	Bioneer	K-3032
AccuRapid™ TA Cloning Kit	Bioneer	K-7170
Dual-Glo® luciferase reporter assay kit	Promega	E1910
FavorPrep GEL/PCR Purification Mini kit	FAVORGEN	FAGCK 001-1
KAPA SYBR FAST qPCR Master Mix (2×) kit	KAPA Biosystem	KK4602
Q5® Site-Directed Mutagenesis Kit	New England Biolabs (NEB)	E0554
**Recombinant RNA/DNA**		
lentiCRISPR v2	Addgene	Plasmid #52961
CRISPR sgRNA: AMPKα1	Designed by http://rgenome.net/cas-designer/	GAA GAT CGG CCA CTA CAT TC
CRISPR sgRNA: AMPKα2	Designed by http://rgenome.net/cas-designer/	CTA CGT GCT GGG CGA CAC GC
CRISPR sgRNA: LATS1	Designed by http://rgenome.net/cas-designer/	GCA GCC ATC TGC TCT CGT CG

CRISPR sgRNA: LATS2	Designed by http://rgenome.net/cas-designer/	GTA GGA CGC AAA CGA ATC GC
CRISPR sgRNA: LKB1	Designed by http://rgenome.net/cas-designer/	CGC CGC AAG CGG GCC AAG CT
CRISPR sgRNA: TSSK1B	Designed by http://rgenome.net/cas-designer/	CAA GAT CAT CGA CCG CAA GA
**Oligonucleotides**		
siRNA: Yap	Dharmacon	J-046247-10
siRNA: Taz	Dharmacon	J-041057-10
Non-targeting scrambled siRNA duplex	Dharmacon	D-001810-01
shRNA: TSSK1B	Merck	TRCN0000219667
shRNA: TSSK1B	Merck	TRCN0000219668
T174A primers for site-directed mutagenesis	Designed by NEBaseChanger	F: ATG GGG CCT ACC ATA CGA<br>R: AGA AAA ATG GTC CTT TGT ACT TGT GGG
Human *CTGF* qPCR primer	Ref. (1)	F: TGG TGC AGC CAG AAA GCT C<br>R: CCA ATG ACA ACG CCT CCT G
Human *CYR61* qPCR primer	Ref. (1)	F: AGC CTC GCA TCC TAT ACA ACC<br>R: TTC TTT CAC AAG GCG GCA CTC
Human *GAPDH* qPCR primer	Designed by Primer Express™ Software v3.0.1	F: CCT GCA CCA CCA ACT GCT TA<br>R: GGC CAT CCA CAG TCT TCT GAG
Human *HPRT1* qPCR primer	Ref. (1)	F: AGA ATG TCT TGA TTG TGG AAG A<br>R: ACC TTG ACC ATC TTT GGA TTA
Human *TSSK1B* #1 qPCR primer	Designed by Primer Express™ Software v3.0.1	F: GA AGT CGG ACA GCT TGA TG<br>R: AGT ACT GCC ACG ACC TGG AC
Human *TSSK1B* #2 qPCR primer	Designed by Primer Express™ Software v3.0.1	F: GAT GCT GCG TAT CCA GAA GGA G<br>R: TGG CTG AGG ATC TCG TCG ATG T
Human *ANKRD1* qPCR primer	Ref. (2)	F: CAC TTC TAG CCC ACC CTG TGA<br>R: CCA CAG GTT CCG TAA TGA TTT
Human *EDN1* qPCR primer	Designed by Primer Express™ Software v3.0.1	F: TGT GTC TAC TTC TGC CAC CT<br>R: CCC TGA GTT CTT TTC CTG CTT
**Cell line**		
A375P	Ref. (3)	10.1128/MCB.02372-06
HEK293A	Ref. (1)	10.1038/ncb3111
HEK293T	Ref. (1)	10.1038/ncb3111
MEF Lats1/2 KO	Ref. (4)	10.1038/emboj.2013.102
U2OS	Obtained from Dr. Kun-Liang Guan's laboratory at the University of California San Diego	
MCF10A		
MCF7		
OCM2.3		
OCM8		
M14		
TM4	KCLB 21715	Korean Cell Line Bank
RT4	KCLB 30002	Korean Cell Line Bank
T47D	KCLB 30133	Korean Cell Line Bank
HCT116	KCLB 10247	Korean Cell Line Bank

**Experimental Models**		
BALB/c nude mouse	Orientbio Inc	
**Software**		
GraphPad Prism 8	GraphPad	https://www.graphpad.com/scientificsoftware/prism/
Photoshop CS6 Portable	Adobe	
ZEN 3.5 blue edition program	ZEISS	
AlphaEaseFC	Alpha Innotech	

References

1. Mo JS, Meng Z, Kim YC, Park HW, Hansen CG, Kim S, et al. Cellular energy stress induces AMPK-mediated regulation of YAP and the Hippo pathway. Nat Cell Biol. 2015;17(4):500-10.
2. Moon S, Kim W, Kim S, Kim Y, Song Y, Bilousov O, et al. Phosphorylation by NLK inhibits YAP-14-3-3-interactions and induces its nuclear localization. EMBO Rep. 2017;18(1):61-71.
3. Mo JS, Kim MY, Han SO, Kim IS, Ann EJ, Lee KS, et al. Integrin-linked kinase controls Notch1 signaling by down-regulation of protein stability through Fbw7 ubiquitin ligase. Mol Cell Biol. 2007;27(15):5565-74.
4. Kim M, Kim M, Lee S, Kuninaka S, Saya H, Lee H, et al. cAMP/PKA signalling reinforces the LATS-YAP pathway to fully suppress YAP in response to actin cytoskeletal changes. EMBO J. 2013;32(11):1543-55.
