# Supplementary material for: The LKB1–TSSK1B axis controls YAP phosphorylation to regulate the Hippo–YAP pathway
Source: Cell Death Dis. 2024 Jan 20;15(1):76. doi: 10.1038/s41419-024-06465-4 (PMC10799855; doi:10.1038/s41419-024-06465-4)

Fig. 1A

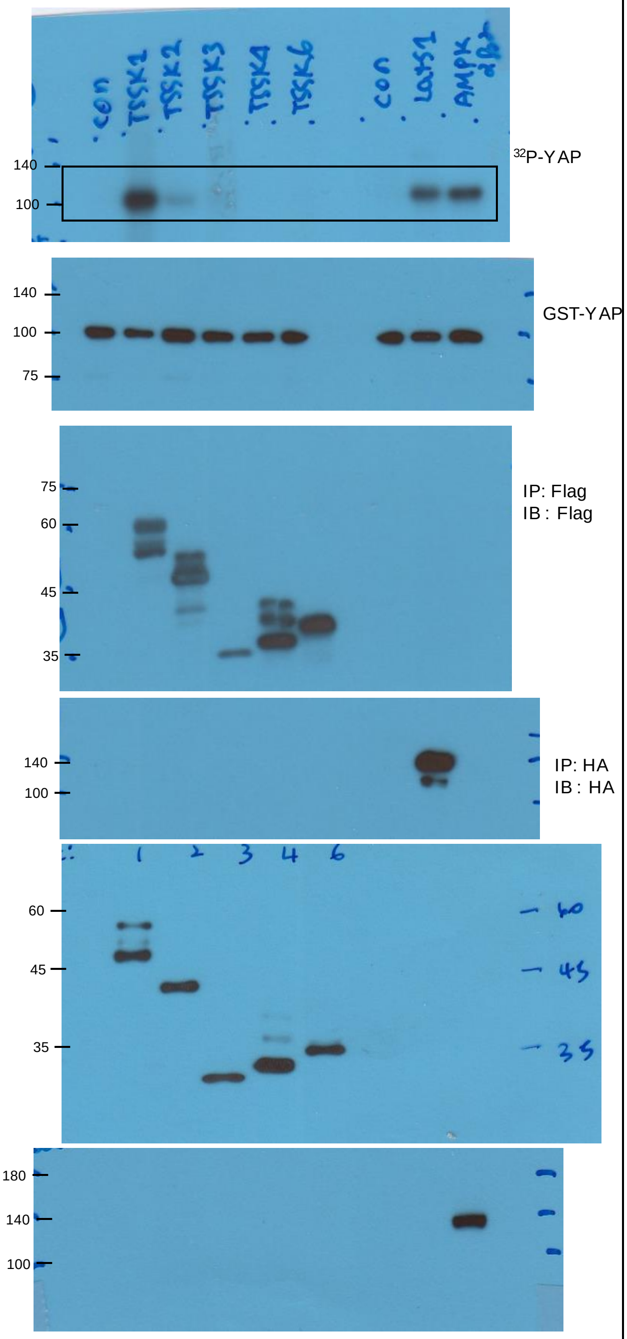

Fig. 1B

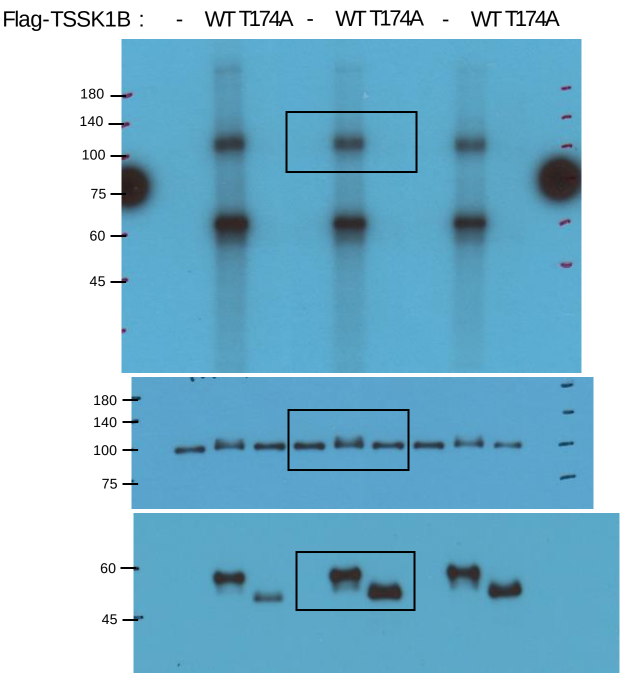

Fig. 1C

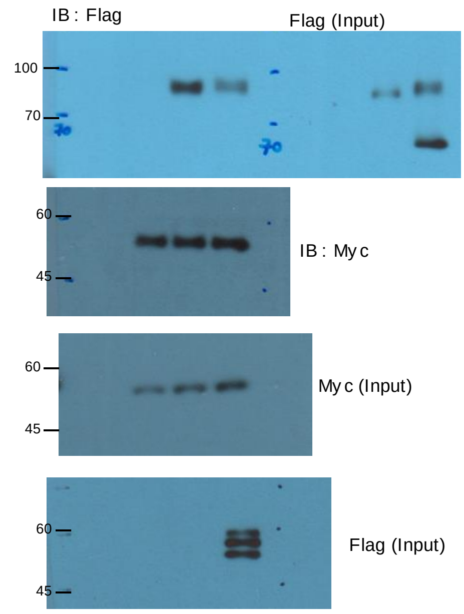

Fig. 2B

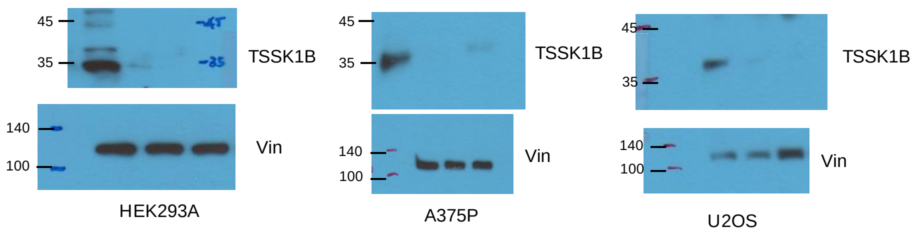

Fig. 2E

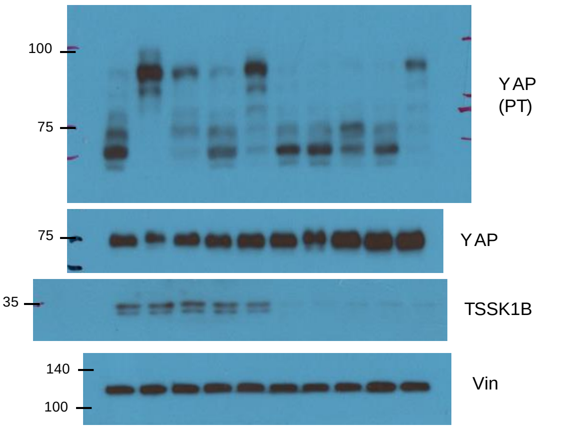

Fig. 2F

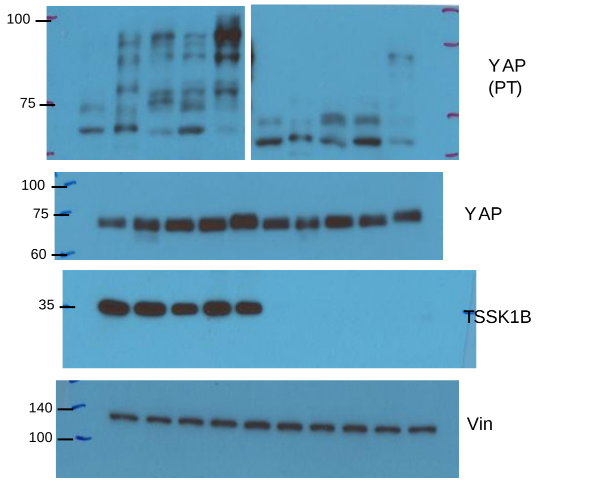

Fig. 3A

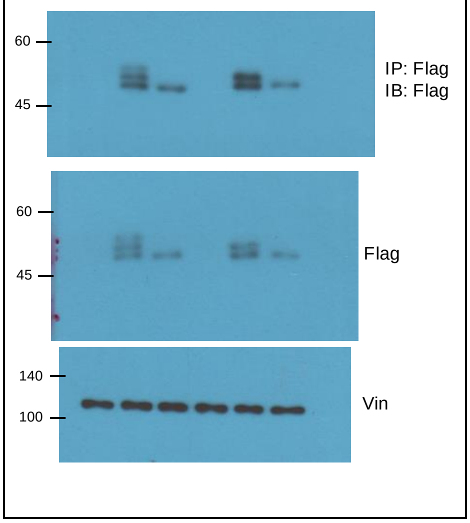

Fig. 3B

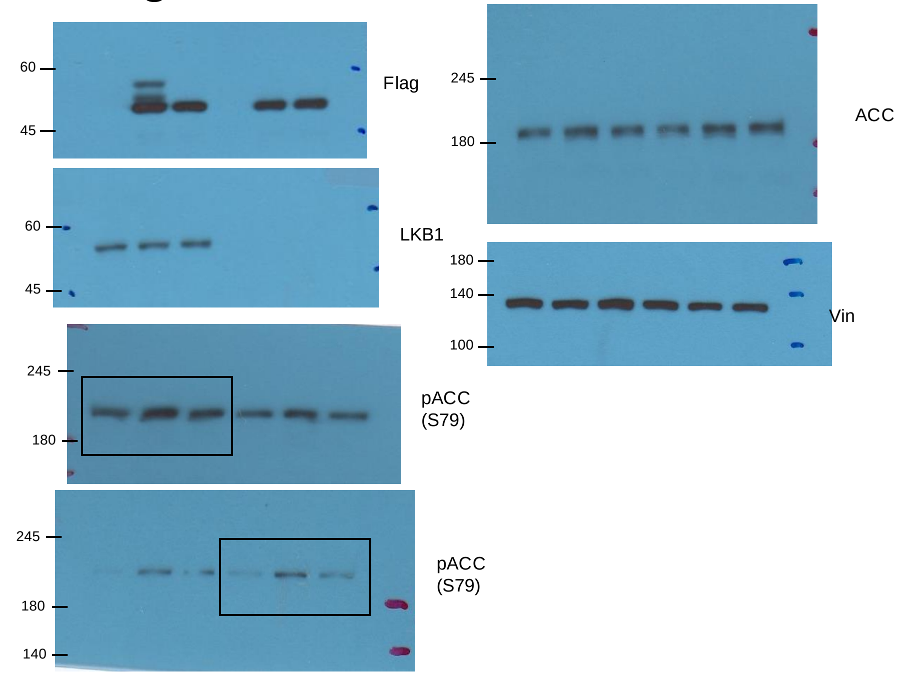

Fig. 3C

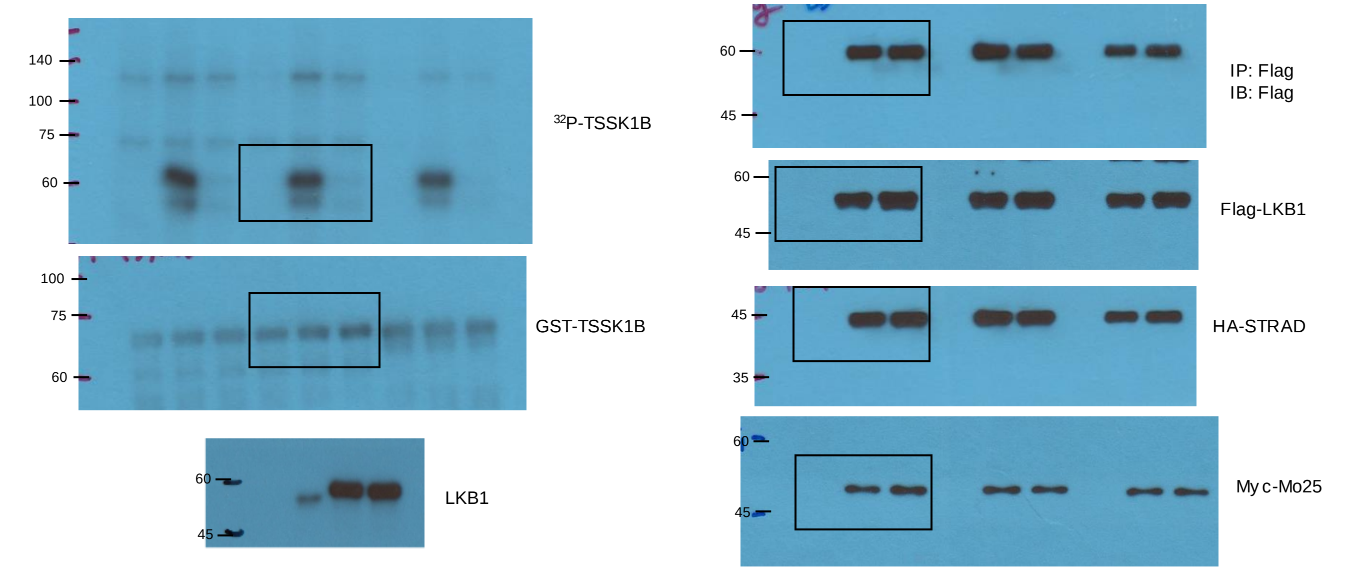

Fig. 3F

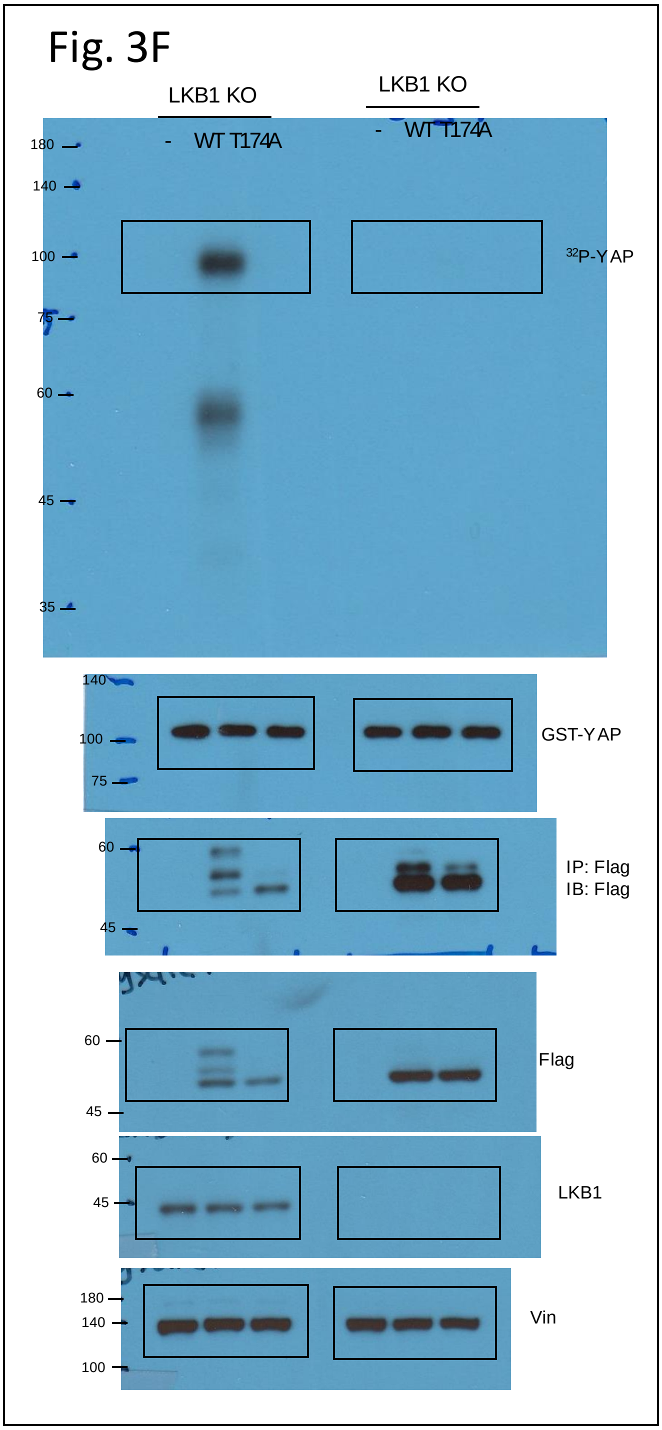

Fig. 3G

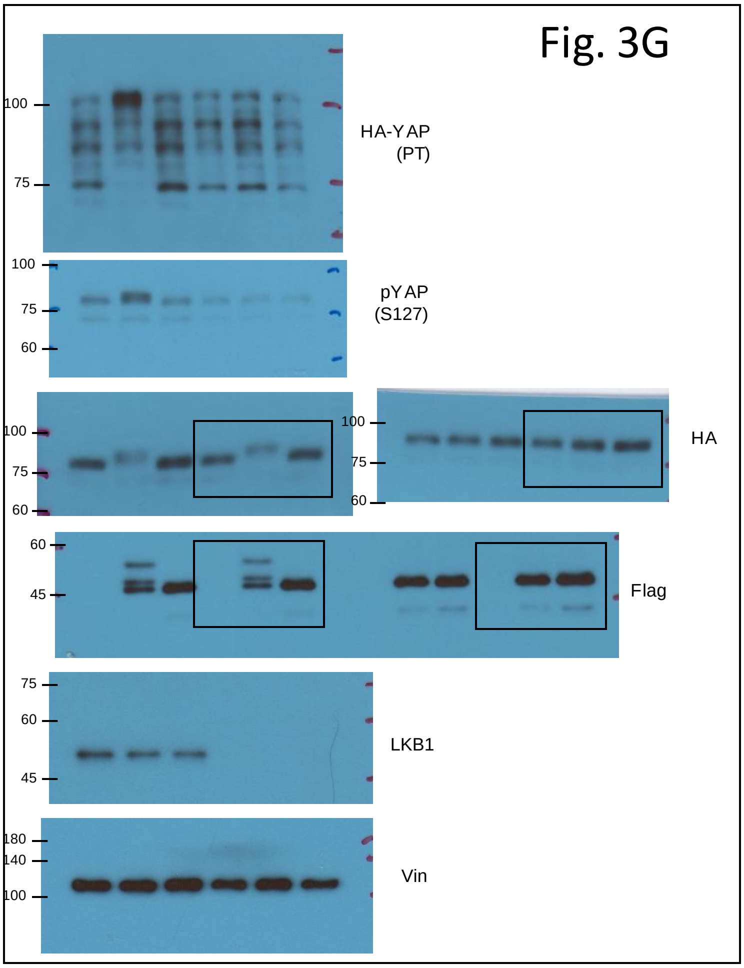

Fig. 4B

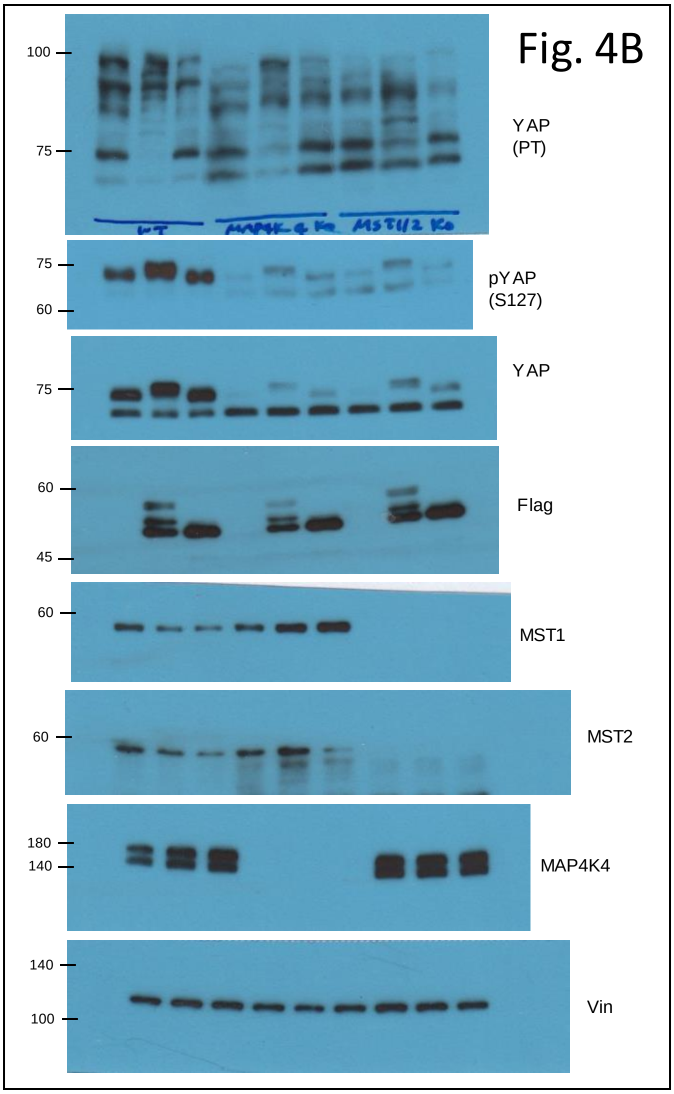

Fig. 4A

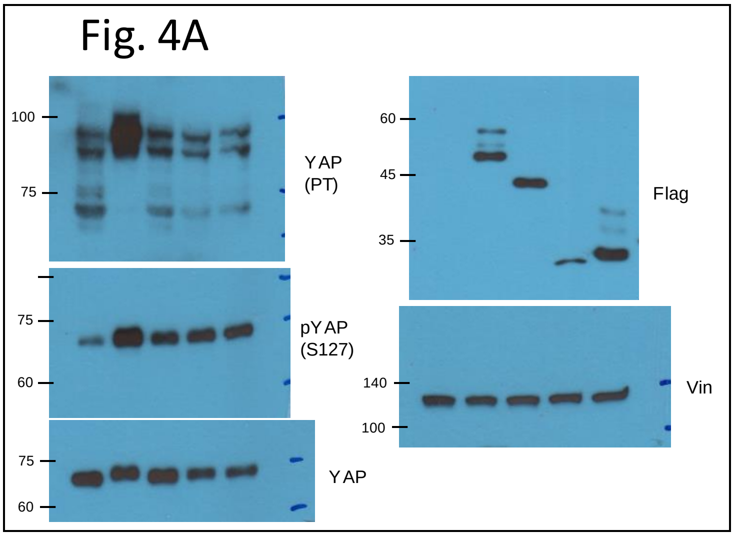

Fig. 4C

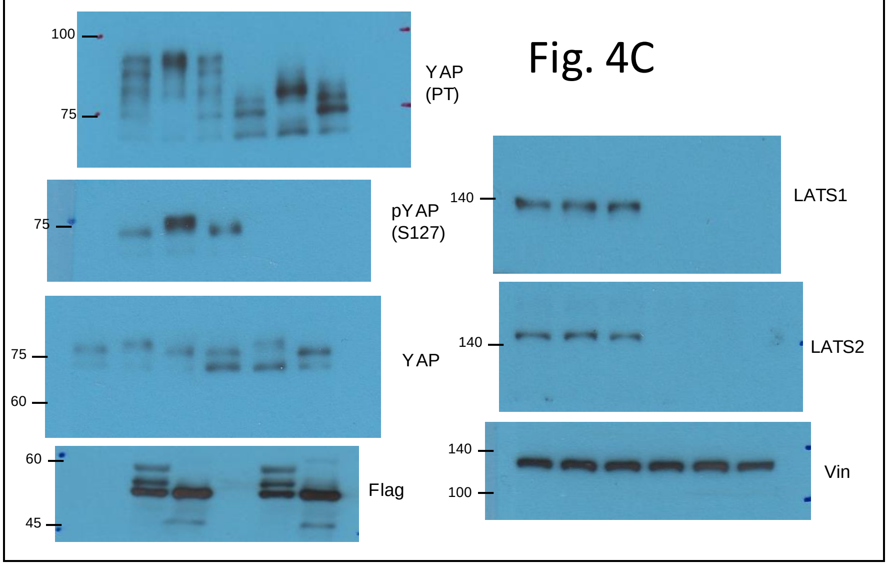

Fig. 4D

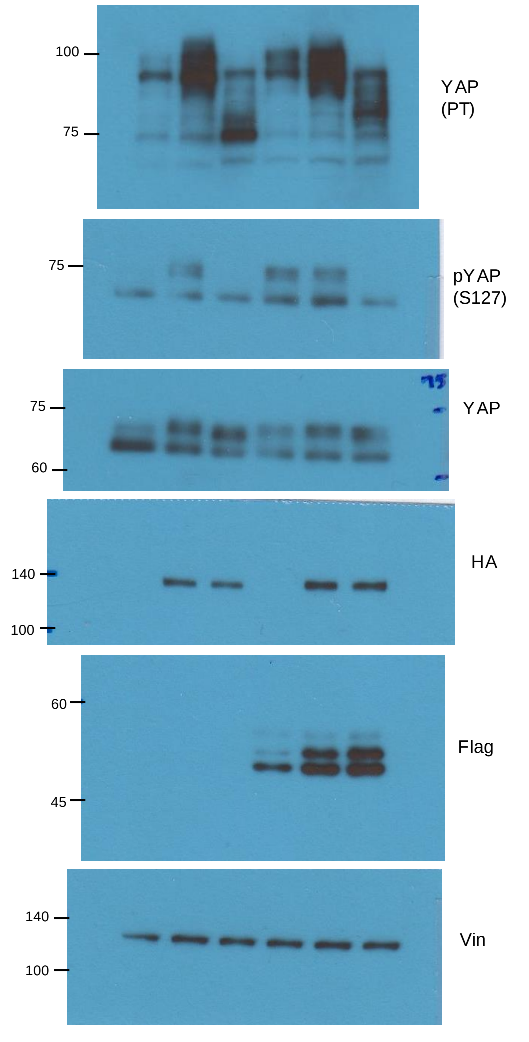

Fig. 4E

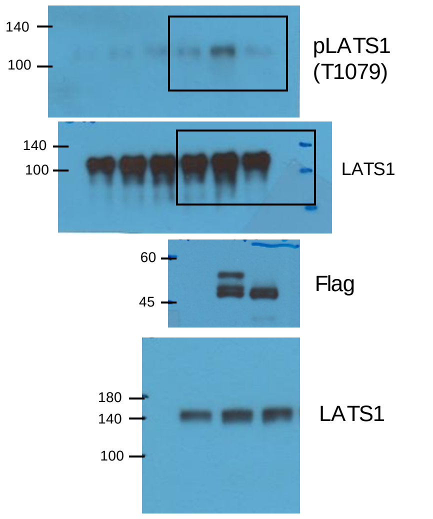

Fig. 5A

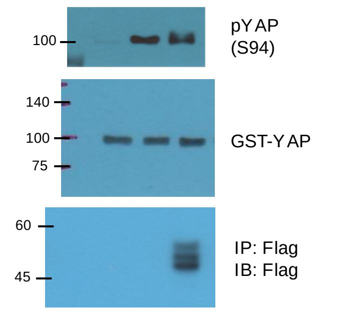

Fig. 5C

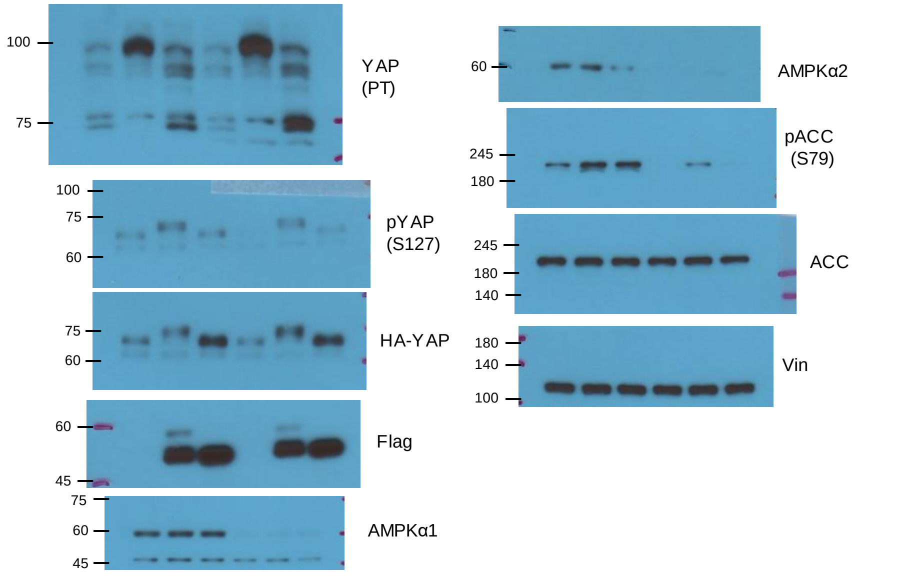

Fig. 5D

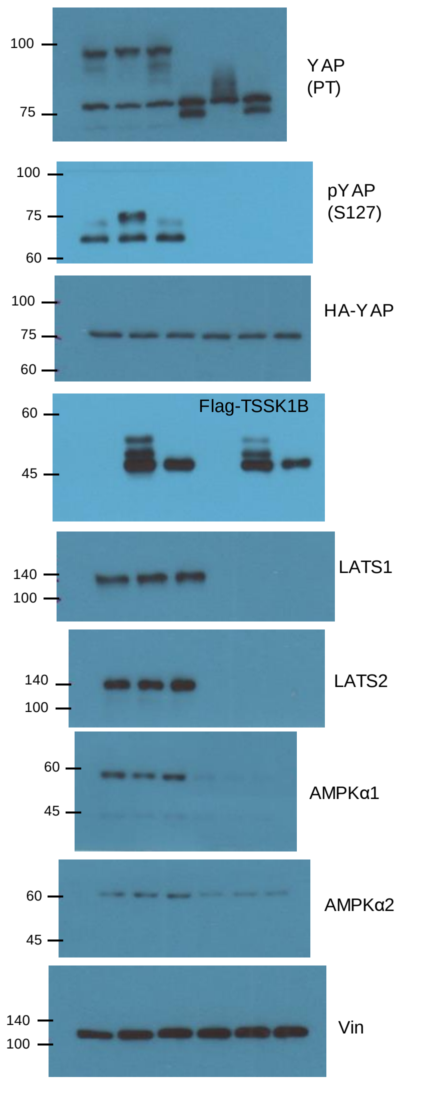

Fig. 5E

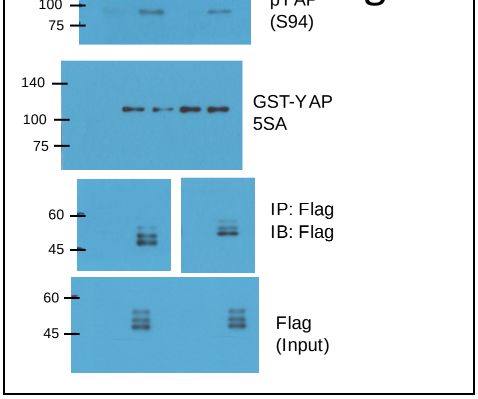

Supplementary Fig. 1A

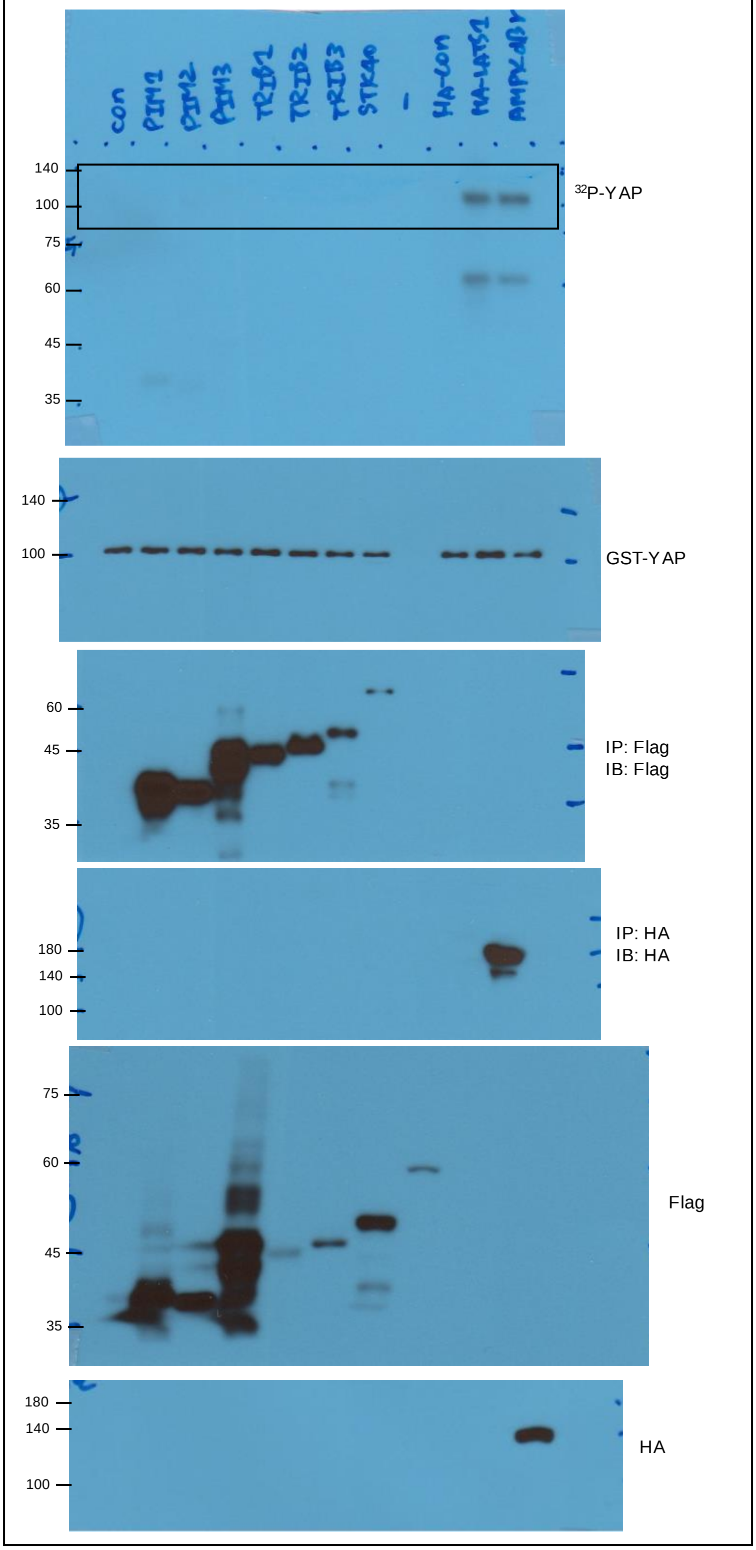

Supplementary Fig. 2A

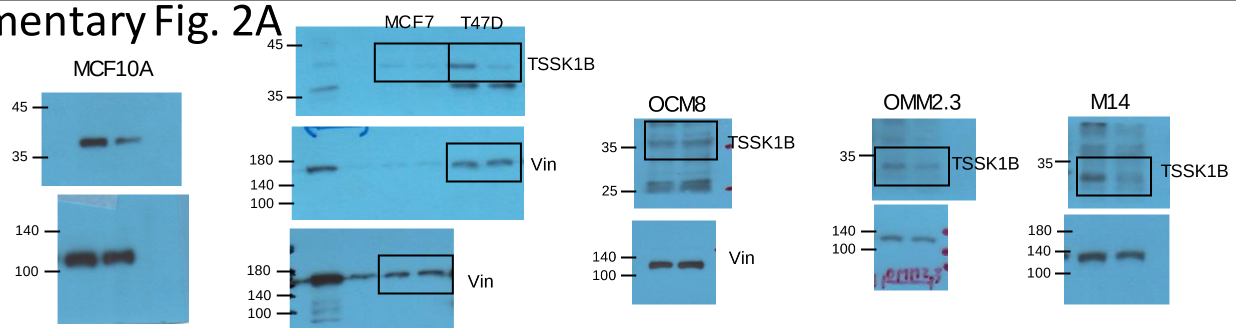

Supplementary Fig. 2A

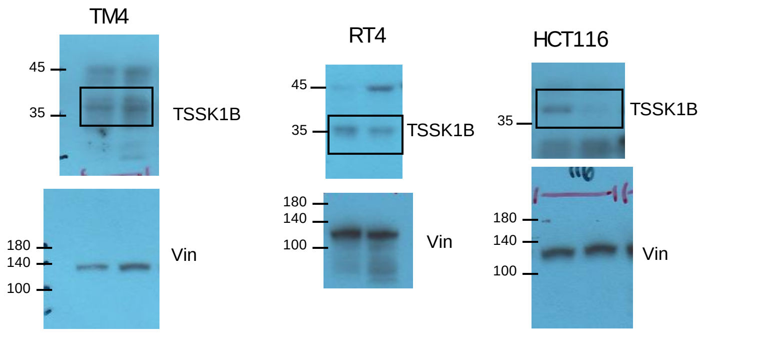

Supplementary Fig. 3A

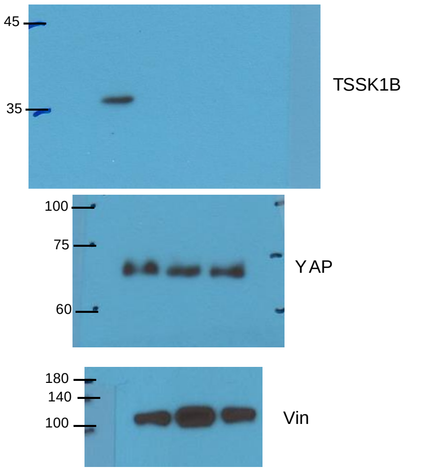

Supplementary Fig. 4B

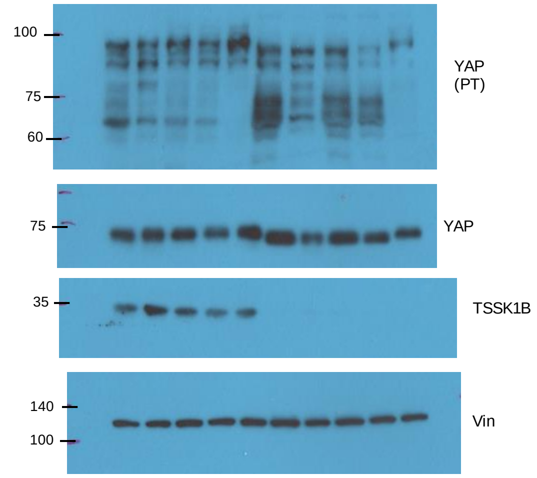

Supplementary Fig. 5B

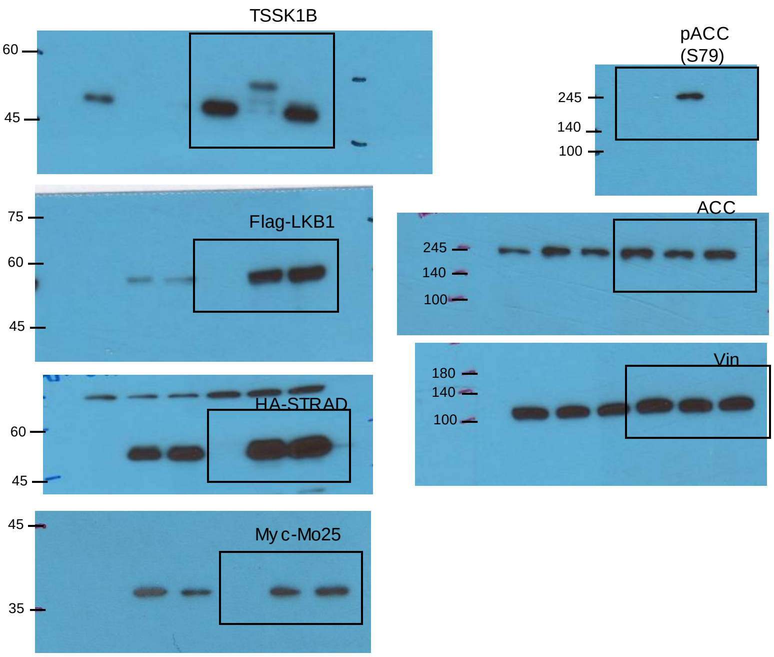

Supplementary Fig. 5C

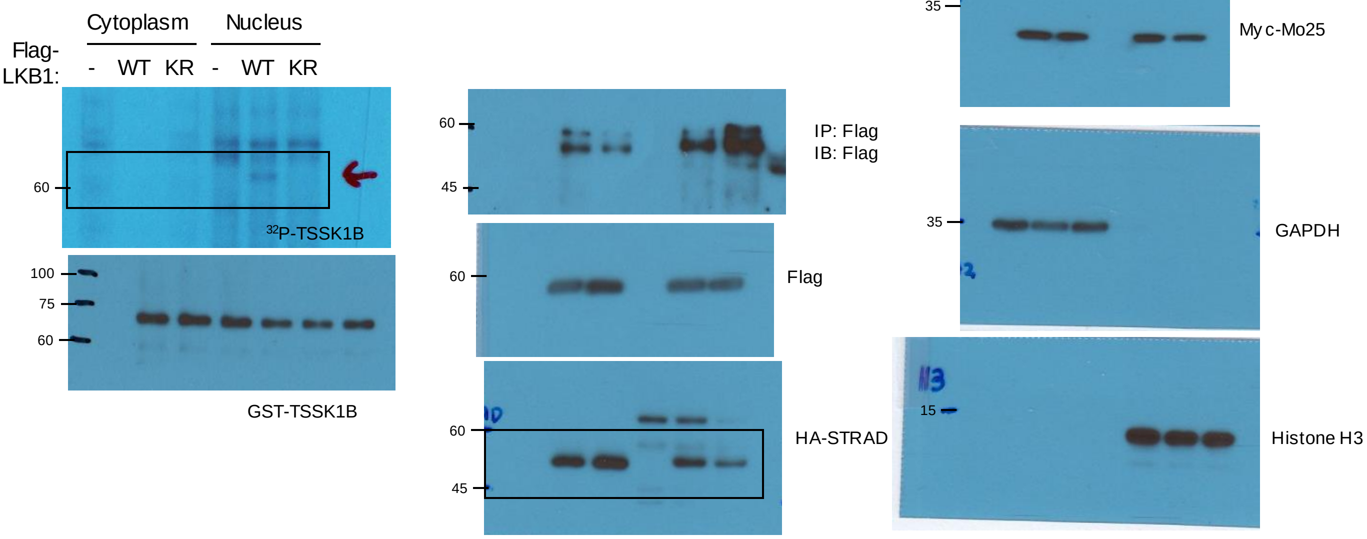

Supplementary Fig. 5D

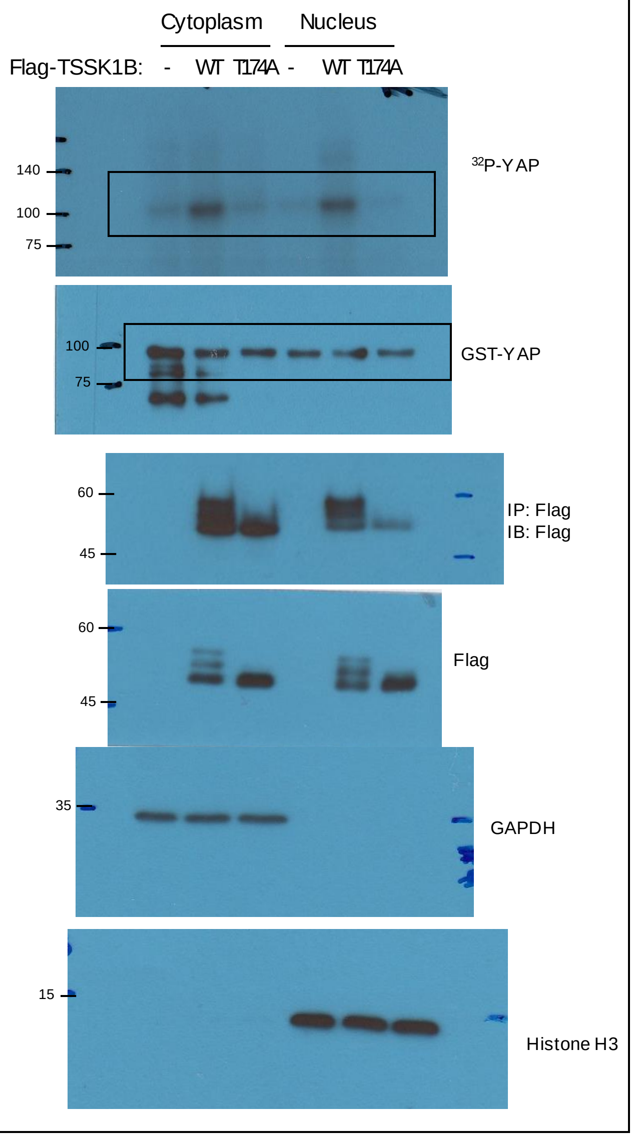

Supplementary Fig. 6A

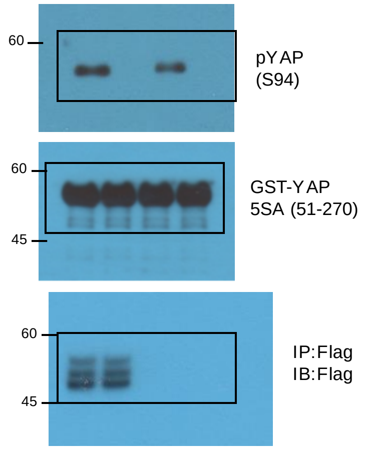

Supplementary Fig. 6B

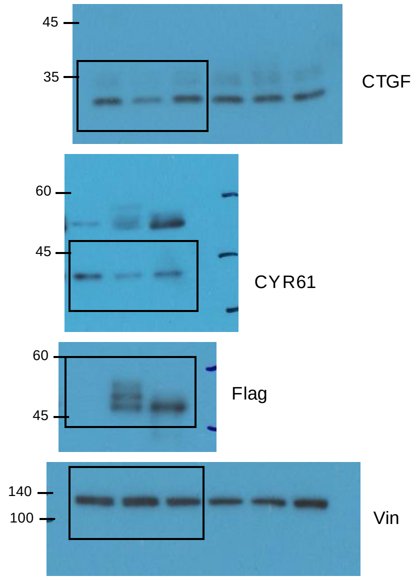

Supplementary Fig. 6D

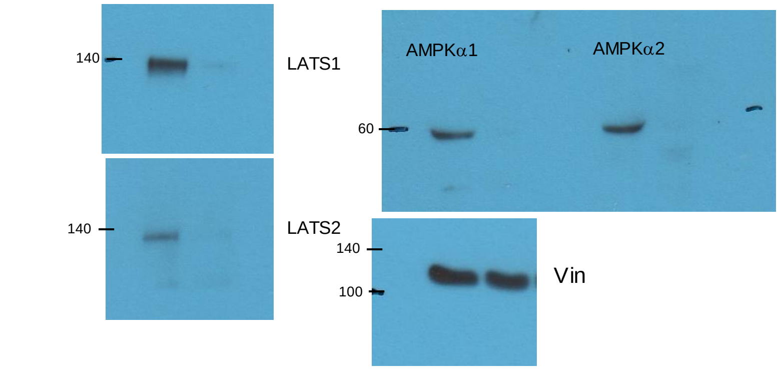

Supplementary Fig. 7A

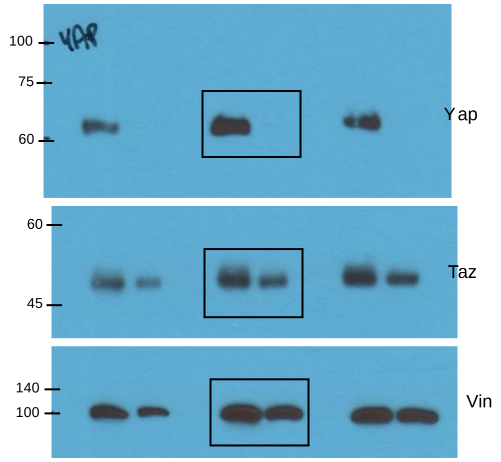

Supplementary Fig. 7B

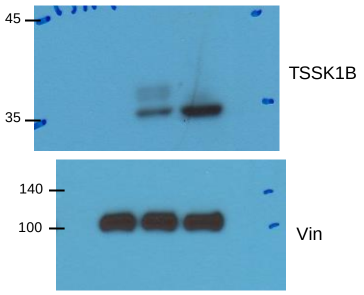

Supplementary Fig. 7C

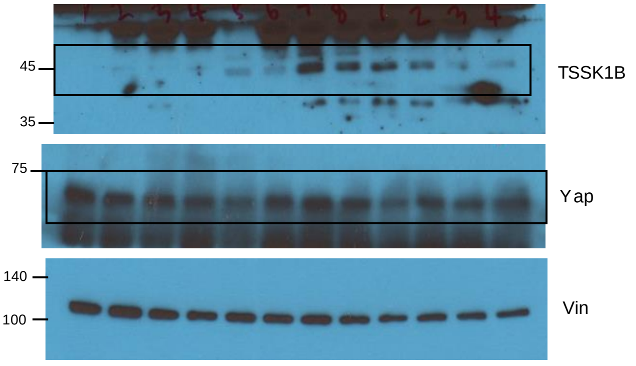

Supplement: Supplementary file 2 — Original Data File [file 41419_2024_6465_MOESM2_ESM.pdf]
